# A Multi-Modal MRI Analysis of Cortical Structure in Relation to Gender Dysphoria, Sexual Orientation, and Age in Adolescents

**DOI:** 10.3390/jcm10020345

**Published:** 2021-01-18

**Authors:** Malvina N. Skorska, Sofia Chavez, Gabriel A. Devenyi, Raihaan Patel, Lindsey T. Thurston, Meng-Chuan Lai, Kenneth J. Zucker, M. Mallar Chakravarty, Nancy J. Lobaugh, Doug P. VanderLaan

**Affiliations:** 1 Child and Youth Psychiatry Division, Centre for Addiction and Mental Health, Toronto, ON M6J 1H4, Canada; malvina.skorska@utoronto.ca (M.N.S.); mengchuan.lai@utoronto.ca (M.-C.L.); 2 Brain Health Imaging Centre, Centre for Addiction and Mental Health, Toronto, ON M5T 1R8, Canada; sofia.chavez@utoronto.ca (S.C.); nancy.lobaugh@gmail.com (N.J.L.); 3 Department of Psychiatry, Temerty Faculty of Medicine, University of Toronto, Toronto, ON M5T 1R8, Canada; kzucker.phd@gmail.com; 4 Cerebral Imaging Centre, Douglas Mental Health University Institute, Montreal, QC H4H 1R3, Canada; gabriel.devenyi@mcgill.ca (G.A.D.); patelraihaan@gmail.com (R.P.); mallar.chakravarty@douglas.mcgill.ca (M.M.C.); 5 Department of Psychiatry, McGill University, Montreal, QC H3A 1A1, Canada; 6 Department of Biological and Biomedical Engineering, McGill University, Montreal, QC H3A 2B4, Canada; 7 Department of Psychology, University of Toronto Mississauga, Mississauga, ON L5L 1C6, Canada; lindsey.thurston@utoronto.ca; 8 The Margaret and Wallace McCain Centre for Child, Youth & Family Mental Health, Azrieli Adult Neurodevelopmental Centre, and Campbell Family Mental Health Research Institute, Centre for Addiction and Mental Health, Toronto, ON M6J 1H4, Canada; 9 Department of Psychiatry and Autism Research Unit, the Hospital for Sick Children, Toronto, ON M5G 1X8, Canada; 10 Autism Research Centre, Department of Psychiatry, University of Cambridge, Cambridge CB2 8AH, UK; 11 Department of Psychiatry, National Taiwan University Hospital and College of Medicine, Taipei 100229, Taiwan; 12 Department of Medicine, Division of Neurology, Temerty Faculty of Medicine, University of Toronto, Toronto, ON M5S 3H2, Canada

**Keywords:** gender dysphoria, cortical thickness, cortical surface area, T1 relaxometry, sexual orientation, adolescence, brain development, brain tissue microstructure, partial least squares, structural MRI

## Abstract

Gender dysphoria (GD) is characterized by distress due to an incongruence between experienced gender and sex assigned at birth. Sex-differentiated brain regions are hypothesized to reflect the experienced gender in GD and may play a role in sexual orientation development. Magnetic resonance brain images were acquired from 16 GD adolescents assigned female at birth (AFAB) not receiving hormone therapy, 17 cisgender girls, and 14 cisgender boys (ages 12–17 years) to examine three morphological and microstructural gray matter features in 76 brain regions: surface area (SA), cortical thickness (CT), and T1 relaxation time. Sexual orientation was represented by degree of androphilia-gynephilia and sexual attraction strength. Multivariate analyses found that cisgender boys had larger SA than cisgender girls and GD AFAB. Shorter T1, reflecting denser, macromolecule-rich tissue, correlated with older age and stronger gynephilia in cisgender boys and GD AFAB, and with stronger attractions in cisgender boys. Thus, cortical morphometry (mainly SA) was related to sex assigned at birth, but not experienced gender. Effects of experienced gender were found as similarities in correlation patterns in GD AFAB and cisgender boys in age and sexual orientation (mainly T1), indicating the need to consider developmental trajectories and sexual orientation in brain studies of GD.

## 1. Introduction

The *Diagnostic and Statistical Manual of Mental Disorders, 5th edition (DSM-5)* [1] characterizes gender dysphoria (GD) as distress due to an incongruence between a person’s experienced gender and sex assigned at birth. The number of adolescents referred clinically for GD has steadily increased over the past two decades [2,3], with a recent population prevalence estimate of 0.5% in adolescents and adults [4]. Reports indicate a recent reversal in the sex ratio of clinic-referred adolescents, with more adolescents experiencing GD assigned female at birth (GD AFAB) than assigned male at birth (GD AMAB) [2,3]. Despite this increase in GD adolescents, and in particular GD AFAB, the neural basis of GD development remains poorly understood. The current study provides insight by examining three structural brain features—cortical thickness, surface area, and T1 relaxation time—in GD AFAB adolescents not receiving puberty blockers or hormone therapy.

One prior study examined cortical and subcortical gray matter volume in GD individuals ages 10 to 21 years [5] and included GD participants not receiving hormone therapy as well as those receiving puberty blockers or gender-affirming hormone therapy. (Given our focus on GD AFAB not receiving puberty blockers or hormone therapy, we do not report the results of Hoekzema et al. [5] from their sample of GD adolescents receiving puberty blockers or hormone therapy.) GD AFAB (*n* = 17) and GD AMAB (*n* = 11) not receiving puberty blockers or hormone therapy generally had cortical and subcortical gray matter volumes similar to individuals of the same sex assigned at birth. In a small number of regions displaying sex differences, gender identity differences were also found: in GD AFAB, a smaller left superior medial frontal cortex volume vs. cisgender (i.e., experienced gender and sex assigned at birth align) girls (*n* = 52), and in GD AMAB, a smaller volume in the left superior posterior hemisphere of the cerebellum and marginally smaller hypothalamic volumes vs. cisgender boys (*n* = 44). Hoekzema et al. [5] concluded that their findings supported the idea that some brain regions in GD adolescents develop in the direction of the experienced gender.

The neurohormonal hypothesis is the most commonly invoked theoretical framework for explaining patterns of brain development in GD [6,7,8,9,10,11]. It posits that prenatal androgen exposure organizes the brain, including the organization of neural sex differences that influence sex-differentiated psychological and behavioral characteristics, such as gender identity and sexual orientation. The surge in sex hormones during adolescence influences brain regions to be further expressed in a relatively male- or female-typical manner, based on the earlier prenatal brain organization [12].

Consistent with this framework, adolescent sex differences in cortical structure have been reported. Cisgender adolescent boys have been shown to have larger surface area than cisgender adolescent girls [13,14,15]. Evidence for sex differences in cortical thickness is more mixed, with some studies finding males have thicker cortices [13] and others finding no differences [14,15]. Sex differences in measures sensitive to underlying microstructure, such as T1 relaxation time, have either not been assessed or have not been observed [16,17,18]. However, a study in 8- to 23-year-olds of gray matter density, a value resulting from soft segmentation of T1-weighted (T1w) images and considered to indicate the amount of gray matter in a given voxel, found higher mean values of gray matter density in females than in males in conjunction with smaller regional cortical volumes [19]. One interpretation was that this finding reflected reduced neuronal density and increased neuropil mass in females, consistent with stereologic morphometry findings [20]. Gray matter density calculations rely on T1w contrast, which is predominantly driven by T1 relaxation time (T1), suggesting that maps of T1 (T1 maps) could be sensitive to sex- and gender-related tissue microstructure differences.

In light of the neurohormonal hypothesis and these observations concerning adolescent brain sex differences, adolescents who experience GD might show cortical surface area, thickness, and T1 features consistent with the experienced gender. An important consideration in evaluating this prediction is that puberty blocking and gender-affirming hormone therapies are often prescribed to ameliorate the distress associated with GD and to promote social transition, including development of the desired physical appearance [11,21]. Because hormone therapy may also impact adolescent brain development [12], it is critical to test predictions about brain organization in GD prior to the onset of hormone therapy [6].

The neurohormonal hypothesis has also been invoked to explain the development of sexual orientation [22,23,24,25], but sexual orientation has seldom been investigated in brain studies of GD ([6]; but see [26] for a study in adults). For example, Hoekzema et al. [5] did not examine associations with sexual orientation, likely because all GD AFAB were gynephilic (i.e., attracted to girls/women) and the GD AMAB were mostly androphilic (i.e., attracted to boys/men). As such, sexual orientation could be an important variable to examine in relation to brain features associated with GD [6].

Adolescence is a dynamic time for sexual orientation development. Retrospective reports indicate that sexual attractions first occur around age 10 for cisgender boys and girls [27,28,29], and often before any first sexual behaviors are experienced [30,31]—although some degree of instability in sexual attractions during adolescence exists, suggesting ongoing exploration with sexuality [32,33]. Developmentally, the strength of sexual attractions, independent of the target, is also an important variable to consider. For example, intensity of sexual behaviors increased (i.e., from hugging to having sexual intercourse) with age in adolescents, and the likelihood of engagement in same-sex behaviors increased in later adolescence [34].

The brain undergoes many changes during adolescence. Cortical volume, which can be decomposed into cortical thickness and surface area, is mostly established by approximately age five and is largely reflected by the development of surface area [13]. There is a surge in the growth of surface area in the first year of life followed by more gradual growth until childhood or adolescence, whereas cortical thickness peaks by approximately age four or five and then slowly declines throughout adolescence and into adulthood [13,35,36,37]. Furthermore, surface area is reported to be influenced by different genetic factors than those involved in cortical thickness [38,39] and surface area is affected more by early prenatal environment factors than cortical thickness [40,41]. During adolescence, the commonly observed decreases in cortical thickness are partially attributable to increases in cortical myelination, which continues well into adulthood [42]. T1 can be a sensitive marker of these myelinating processes, including during adolescence [17,18,43,44,45,46].

To investigate associations between cortical structure and GD, the present study examined cortical gray matter in GD AFAB adolescents not receiving hormone therapy relative to cisgender girls and boys. Using the multivariate partial least squares (PLS) approach, we examined three structural brain features (cortical thickness, surface area, and T1) to characterize patterns across 76 brain regions. The primary aim was to identify multivariate brain patterns that distinguished the three groups. A secondary aim was to evaluate group differences in multivariate brain patterns related to sexual orientation and age. Two aspects of sexual orientation were examined: degree of androphilia-gynephilia, and strength of sexual attractions. In line with the prior studies reviewed above, we predicted that there would be sex differences in surface area, cortical thickness, and/or T1 between cisgender boys and girls [13,14,15,19,20]. GD AFAB not receiving puberty blockers or hormone therapy were expected to differ from cisgender girls and to be similar to cisgender boys on these cortical structural metrics [5]. Based on prior findings, it was predicted that this would especially apply to surface area (e.g., [15,16]). Furthermore, the cortical features of gynephilic GD AFAB should especially reflect those of gynephilic cisgender boys [6].

## 2. Materials and Methods

### 2.1. Participants

A total of 47 adolescents participated in the study from 2014 to 2018: 16 AFAB adolescents with a DSM-5 diagnosis of GD not receiving hormone therapy, 17 cisgender adolescent girls, and 14 cisgender adolescent boys. Participants were 12 to 17 years old, with a mean age of 15.32 years (*SD* = 1.64). The majority were “European”/“White” (*n* = 27, 57.4%) and the remainder were other ethnicities (*n* = 20, 42.6%) (see Supplementary Materials for more information). Data from four additional participants were removed: one GD AFAB adolescent due to artifacts from a metal orthodontic expander, one cisgender boy due to excessive head motion, and one GD AFAB adolescent and one cisgender boy due to unusable images needed to calculate the T1 maps.

Inclusion criteria for the study were age 12 to 17 years. GD AFAB participants had to have received a diagnosis of GD via clinician assessment. Exclusion criteria were: receiving any form of hormone therapy (apart from oral contraceptive pills), a known disorder of hormone regulation or sex development, any contraindications to magnetic resonance imaging (MRI, e.g., braces, pregnancy), experience of any head trauma, or insufficient English language proficiency to complete study measures. For cisgender individuals, additional exclusion criteria were: a previous mental health diagnosis, being in a special education class in school, involvement with a child protection agency, and feeling uncomfortable with their sex assigned at birth and identifying or wishing to identify as a member of another gender.

GD AFAB participants were recruited from the Gender Identity Service at the Centre for Addiction and Mental Health or were referred by a clinician in private practice specializing in GD. At the Gender Identity Service, all potential participants within the requisite age range were informed of the opportunity to participate in the study. If they were interested in doing so, a member of the study team, who was not involved in their clinical care, shared information about the study, answered questions, performed screening, and obtained informed assent and/or consent to participate. Cisgender participants were recruited from the community via advertisements describing the study as being about brain development and gender. Advertisements were posted online (Kijiji, Facebook) and on bulletin boards in the Greater Toronto area (e.g., City of Toronto message boards). Study information was also distributed to cisgender participants via word of mouth to facilitate snowball sampling.

### 2.2. Procedures

Prospective participants were screened for eligibility in person or over the phone. If eligible, participants between 12 and 15 years old provided verbal assent and a parent/guardian provided informed consent. Participants 16 to 17 years old provided informed consent. After enrolment into the study, about 20 mL of blood was drawn to measure hormones and other indices of physiological functioning, a 1-h MRI session was conducted, and participants completed a brief intelligence assessment and questionnaire package. If GD participants completed any study measures during a clinical assessment, these data were obtained from their clinical record. For most participants, all study procedures were completed on the same day, and the order was allowed to vary. At the end of the study visit, participants were thanked and received an honorarium of $20 CAD per hour or part thereof for their time and were also compensated for travel expenses. Only measures relevant to the aims of this study are reported.

### 2.3. Measures

All numerical values for variables reported and most of the code used are available from Scholar’s Portal Dataverse [47]. For measures where a mean is calculated, the mean was computed for those participants who responded to at least 75% of the items.

#### 2.3.1. Age

Age in months was calculated by subtracting the date that the consent form was signed from the date of birth and was rounded to the nearest month. The MRI scan was conducted on the same day as consent for 42 participants, within one month for four participants, and within two months for one participant.

#### 2.3.2. Gender Dysphoria

The Gender Identity/Gender Dysphoria Questionnaire for Adolescents and Adults (GIDYQ-AA) [48,49] is a 27-item questionnaire assessing concurrent gender identity and gender dysphoria with good discriminant validity and clinical utility [47,48,49,50]. Cisgender boys completed the male version and cisgender girls and GD AFAB completed the parallel female version. Each item was rated on a 5-point Likert-type scale ranging from 1 (always) to 5 (never) with respect to the past 12 months. An example of a female version item is: “In the past 12 months, have you felt unhappy being a girl?” A mean score was calculated, and lower scores indicated stronger gender dysphoria.

#### 2.3.3. Sexual Orientation

The Erotic Response and Orientation Scale (EROS) is a 16-item self-report measure assessing sexual orientation with regard to attractions and fantasies over the past 6 months with good discriminant validity [51,52,53]. Half of the questions pertain to attractions/fantasies toward boys/men (i.e., androphilia; e.g., “How often have you noticed you had any sexual feelings [even the slightest] while looking at a boy/man?”). The other half used similar language to assess attractions/fantasies toward girls/women. Frequency of occurrence for each item was rated on a 5-point scale ranging from 1 (not at all) to 5 (almost every day). Mean androphilia and mean gynephilia scores were derived for each participant, where higher scores reflected more attractions/fantasies. Internal consistency on both scales was high (Cronbach’s alpha: androphilia = 0.94, gynephilia = 0.95).

EROS scores measure two aspects of sexual orientation: strength of attractions (i.e., none to many attractions, regardless of orientation) and degree of androphilia-gynephilia. To characterize these two aspects, two metrics were created for each participant. A vector was created by calculating the strength (magnitude) and degree (phase, *θ*) of sexual attractions from the individual androphilia and gynephilia scores, which were not significantly correlated with each other (see Results). Androphilia was arbitrarily placed on the *x*-axis and gynephilia on the *y*-axis. Based on EROS scores (ranging from 1 to 5 on each scale), the strength (magnitude) of sexual attractions was denoted by the length of the vector, calculated for each participant using the following formula:

$$\sqrt{\left({\left(androphilia\;score\right)}^{2}+\;{\left(gynephilia\;score\right)}^{2}\right)}$$

Degree of androphilia-gynephilia was calculated for each participant using the following formula:

$$\left(\arccos\left(\frac{androphilia\;score}{magnitude}\right)\right)*\;\left(\frac{180{}^{\circ}}{\pi }\right)$$

**Figure 1:**
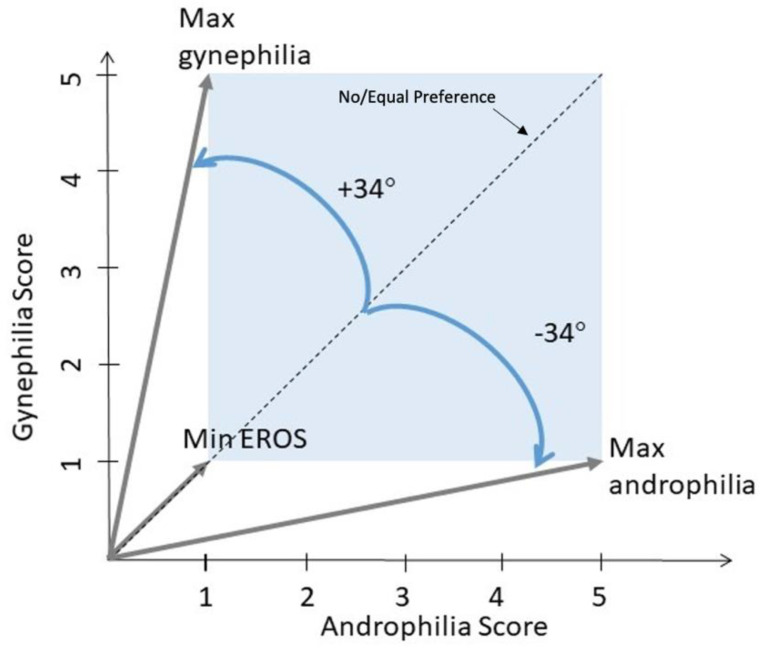
Erotic Response and Orientation Scale (EROS) scoring. The direction (androphilia-gynephilia) and strength of sexual attractions were calculated from the EROS androphilia and gynephilia scores. In the case of equal or no preferences, the vector would lie along the 45° dashed line. If a preference was indicated, the direction could range ±34° from the no preference angle (blue arrows). Maximal androphilia and gynephilia vectors as well as a vector created by minimum responses for both scores are indicated in dark gray. The length of the vector indicates the strength of attractions, independent of the target.

These values are symmetrically distributed ±34° around 45° such that an 11° phase indicates exclusive androphilia, a 79° phase indicates exclusive gynephilia, and a 45° phase indicates equal scores on androphilia and gynephilia (e.g., asexual or ambiphilic) (Figure 1).

### 2.4. Magnetic Resonance Imaging (MRI) Methods

#### 2.4.1. Image Acquisition

All participants were scanned at the Centre for Addiction and Mental Health in Toronto, Ontario, Canada on a GE MR750 3T magnetic resonance scanner (General Electric, Milwaukee, WI, USA) with an 8-channel head coil (General Electric, 8HR BRAIN, GE Standard 8-Channel Head Coil). T1w images were acquired with a T1 BRAVO pulse sequence in the sagittal plane. The acquisition parameters were: inversion time = 650 ms, echo time = 3 ms, repetition time = 6.8 ms, flip angle = 8°, field of view = 23 cm, 256 mm × 256 mm matrix, 200 isotropic 0.9-mm thick slices, acquisition time = 4:42 min. Calculation of the T1 maps with B1 correction required four acquisitions using sagittal spoiled-gradient echo (SPGR) pulse sequences. These scans included: two fast-SPGR scans (repetition time = 10.6 ms, echo time = ~4.4 ms, acquisition time = 2:59 min each) with 1 mm isotropic resolution (field of view = 25.6 cm, 160 slices) at two flip angles (14° and 3°). These two images will be referred to as “Flip14” and “Flip3,” respectively, and were used for T1 mapping calculations using the variable flip angle method [54,55]. Also, to account for B1 inhomogeneities, B1 maps were computed using an extrapolation to signal null as per the method of slopes [56] from two SPGR scans (repetition time = 50–60 ms, echo time = 5 ms, acquisition time = 2:24 min each) with low, 4-mm isotropic resolution (field of view = 25.6 cm, 40 slices) and two flip angles (130° and 150°). Total acquisition time for the four scans was 9:46 min.

#### 2.4.2. T1w Image Processing

T1w images were pre-processed using the minc-bpipe-library on SciNet [57,58]. Briefly, images were realigned to roughly align with the MNI template, signal intensity non-uniformity was corrected [59], and the brain was extracted [60] (see also [61,62]). Extensive quality checking of images for intensity non-uniformity, motion, and quality of brain extraction was performed after pre-processing. The extracted brains were edited manually to remove obvious segmentation errors.

Next, the bias-corrected and brain-extracted T1w images were submitted to the CIVET 2.1.0 processing pipeline [63,64] on SciNet [57,58] to estimate cortical thickness and surface area. Briefly, CIVET extracts a white matter surface (the boundary between cortical gray matter and subcortical white matter) and a pial surface (the boundary between cortical gray matter and cerebral spinal fluid) containing 40,962 vertices per hemisphere. Cortical thickness (mm) is calculated as the distance between the white matter and pial surface at each vertex [65], and surface area (mm^2^) is calculated at the mid-point between the two surfaces using a Voronoi parcellation based on the 6 triangles adjacent to each vertex [66]. A smoothing kernel of 20 mm and 40 mm was applied to statistically regularize the cortical thickness and surface area data, respectively, and the quality of the surface extractions was verified. Mean surface area and mean cortical thickness for 76 regions of interest (ROI) (38 per hemisphere) were computed based on the Anatomical Automatic Labelling (AAL) atlas [67].

#### 2.4.3. T1 Relaxation Time Map Creation

Cortical thickness and surface area are derived from T1w images, but additional, quantitative information regarding subtle cortical microstructural characteristics can be obtained using methods to calculate tissue T1. T1 reflects the rate at which magnetic spins, in a static magnetic field, lose energy and return to their original equilibrium state after absorbing energy by a perturbing time-varying magnetic field. The magnetic spins of interest in brain MRI are the hydrogen nuclei of water molecules and hence, T1 reflects the rate of energy loss, which depends on the interactions of these water molecules with surrounding molecules, including other water molecules, lipids, proteins, and other macromolecules. T1 maps (and extracted T1 values) reflect those interactions at the voxel level and can thus reveal microstructural differences across tissue types and brain regions. Specifically, T1 is shorter in white matter compared with gray matter due to the relative larger numbers of other molecules and fewer free-water molecules (i.e., water that is not in the myelin sheath) [43]. T1 in gray matter becomes shorter with increasing age [44,46], including during adolescence [17,18,45].

All four SPGR images used to create the T1 maps were reoriented into the space defined as halfway between the Flip3 and Flip14 images using FLIRT (FMRIB’s Linear Image Registration Tool) from the FSL (FMRIB Software Library, version 5.0.9) tool library. Specifically, the “halfway flirt” command from FSL’s SIENA pipeline was used. The Flip3 image was brain-extracted using FSL’s BET (Brain Extraction Tool) and the resulting mask was used to extract the brain of the Flip14 image. T1 maps were then calculated and calibrated as described in Chavez [68]. In this procedure, B1 maps were generated using the method of slopes to obtain the true flip angle [56] and T1 maps were computed using the variable flip angle method with a B1 correction incorporated [55].

#### 2.4.4. T1 Map Processing Pipeline

To extract the ROI data from the T1 maps, the reoriented Flip14 image was registered to the brain-extracted T1w image from the bpipe pipeline. The resulting transformation matrix was used to move the T1 map into T1w space using FSL’s FLIRT. Brain-extracted T1w images were registered to the 1-mm MNI template using antsRegistration (version 2.1.0; [69,70]). The resultant matrix was then inverted and used to move the AAL template from MNI space to T1w space using ANTs’ antsApplyTransforms with the generic label option. Each registration step and transformed image was inspected for registration errors.

To better match the cortical thickness data, T1 data were extracted only from gray matter voxels in each ROI. FSL’s Automated Segmentation Tool (FAST) was used to segment the brain-extracted T1w images from the bpipe pipeline into gray matter, white matter, and cerebral spinal fluid, allowing partial volume estimates and disabling the bias field correction. Using an in-house MATLAB (2017b, version 9.3.0.713579 [71]) script, T1 data were extracted for the same 76 AAL atlas ROIs as were used in CIVET. T1 data were included only for voxels where gray matter probability was > 0.40 (i.e., the voxel contained at least 40% gray matter). A histogram of T1 values was plotted for each ROI using T1s 500 ms ≤ T1 ≤ 5000 ms and a bin width of 30 ms. The distributions were generally unimodal (some ROIs included white matter which led to a small peak at lower T1), but were often positively skewed (partly due to partial volume effects between gray matter and cerebral spinal fluid). To avoid overestimating T1 in the ROI, the T1 mode, rather than the mean, was used to represent the T1 value in the ROI. This was accomplished by fitting a spline function to the histogram and finding the T1 value at the peak (see Figure S1 in Supplementary Materials). Histograms of T1 values were spot checked for anomalies.

## 3. Statistical Analyses

### 3.1. Group Differences in Demographic and Psychosexual Variables

Using SPSS version 27 (IBM Corp., 2020), group differences for age, the GIDYQ-AA (gender dysphoria), and EROS (sexual orientation) variables were examined with one-way analyses of variance (ANOVA). In the presence of a significant omnibus effect, post hoc comparisons were conducted with least significant difference (LSD) tests. The GIDYQ-AA had a significant Levene’s test, so the Games-Howell post hoc test, robust to heterogeneity of variance, was used for this measure. A two-tailed critical *p*-value of 0.05 was used.

We also investigated ethnicity, parent education, parent marital status, subtests on the Weschler intelligence scales capturing verbal comprehension and visual spatial indices [72,73,74], pubertal development [75], regularity of menstrual cycle, medication use, externalizing mental health challenges, internalizing mental health challenges, and total mental health challenges [76,77,78]. Tests of group differences for these additional demographic variables are provided in the Supplementary Materials. Also, correlations between these variables and the ROI data are presented, corrected for multiple comparisons. No demographic variable correlated with the ROI data after correction, and age, ethnicity, parent education, verbal comprehension index, visual spatial index, pubertal development, regularity of menstrual cycle, and externalizing mental health challenges did not show significant group differences. Thus, apart from including age as a variable of interest in specific analyses, no other demographic variables were included in the main analyses.

Total brain volume was not included as a covariate because of known sex assigned at birth differences in total brain volume [79], which have been shown to impact detectability of sex differences in cortical thickness [80]. Given that we were interested in examining how GD AFAB adolescents are positioned relative to likely differences between cisgender girls and boys, controlling for total brain volume could overly remove informative sex differences. Furthermore, not all studies have controlled for total brain volume when investigating cortical thickness and surface area (e.g., [13]). Raznahan et al. [13] demonstrated that changes in cortical volume are reflected more in changes in surface area rather than cortical thickness changes, making controlling for total brain volume most relevant for surface area. However, in a recent large-scale analysis of sex differences including surface area, controlling for total brain volume did impact sex differences in cortical thickness and did not impact sex differences in surface area [15]. Given the mixed impact that controlling for total brain volume has had on the detectability of sex differences in surface area and cortical thickness, we chose not to apply this correction.

### 3.2. Partial Least Squares Analyses

The multivariate statistical technique partial least squares (PLS, [81,82]) was used to examine the relationship between the ROI data and group in two analyses. PLS works well with data that are not independent from each other, as well as in situations where the number of observations is larger than the number of participants, both of which are true for brain data. Furthermore, PLS identifies distributed patterns in brain data, including structural MRI data [83]. PLS uses singular value decomposition of a data matrix to produce latent variables with three components: group and/or task contrasts (*v*), brain saliences/weights (*u*), and a measure of the strength of that relationship (*s*). We were primarily interested in identifying the commonalities and differences among the brain metrics as they related to group differences and to group differences in correlation patterns (rather than identifying differences across metrics). To achieve these goals, the data matrix was created as a single row for each participant containing 76 cortical thickness measures, 76 surface area measures, and 76 T1 measures. The results will be referred to by ROI or region “type” (e.g., cortical thickness/surface area/T1). We noted that the distribution of raw surface area values was not normal, but they were for cortical thickness and T1 data. To minimize the impact of the differences in data distributions as well as differences in metric-specific ranges of values on the results, all brain data were normalized by converting them to *z*-scores within each ROI (i.e., across all participants; e.g., [84]).

Permutation tests were used to assess the number of times the strength of the permuted latent variables exceeded the observed strength and provided an exact probability. Bootstrap resampling was used to estimate the standard error of each brain salience, to assess the reliability of their contribution to the observed pattern. The ratio of the salience to its standard error approximates a *z*-score, and for all analyses a threshold of ± 3 was used to identify stable, reliable contributions. Confidence intervals (CI, 95%) around the point estimates were calculated using the bootstrap sampling distribution. For all analyses, 1000 permutations and bootstrap samples were used. For all analyses, “brain scores” were calculated. These scores are the projection (dot-product) of the brain saliences (*u*) with a participant’s data and provide an indication of how strongly each individual reflects the contrast identified on the latent variable.

The first PLS analysis was a “Task PLS,” which in this case, distinguished the three groups based on an optimal combination of surface area, cortical thickness, and T1. Age was not included in this analysis given no group differences in age and given no significant correlations between age and the ROIs after correcting for multiple tests (see also Results and Supplementary Materials). The second analysis was a “Behavior PLS,” which identified the commonalities and differences in brain-behavior correlations across the three groups. For this Behavior PLS analysis, the behavior variables were the two EROS variables (i.e., strength of attractions and degree of androphilia-gynephilia) and age, and the analysis assessed the degree to which they correlated with the brain measures. Age was included because the strength of attractions scores correlated with age (see Results). For the Behavior PLS analysis, the correlations of brain scores with behavior measures, which reflect the observed contrast (*v*), were used [82]. Both PLS analyses were conducted using PLS software (v. 6.1311050) using MATLAB (2014a, version 8.3.0.532, [85]). Correction for multiple comparisons is not required because statistical significance is only evaluated in relation to the strength (*s*) of the latent variable [82].

## 4. Results

### 4.1. Demographic and Psychosexual Variables

Descriptive statistics by group are shown in Table 1. There were no extreme deviations from normality based on skewness and kurtosis values, which were less than |2|.

For age, the main effect of group was not significant (see Table 1). As expected, there was a significant effect for the GIDYQ-AA (gender dysphoria) such that the GD AFAB group scored significantly lower than both the cisgender girls (mean difference (MD) = −2.69, *SE* = 0.10, *p* < 0.001) and cisgender boys (MD = −2.70, *SE* = 0.09, *p* < 0.001), who did not differ from each other (MD = 0.01, *SE* = 0.05, *p* = 0.970). The threshold for a potential diagnosis of GD (“caseness”) on the GIDYQ-AA is ≤3.00 [50]. The lowest score on the GIDYQ-AA from the cisgender participants was 4.48, indicating that none of the cisgender participants met this threshold; the highest score in the GD AFAB group was 3.04, with all other GD AFAB participants scoring below the threshold.

**Figure 2:**
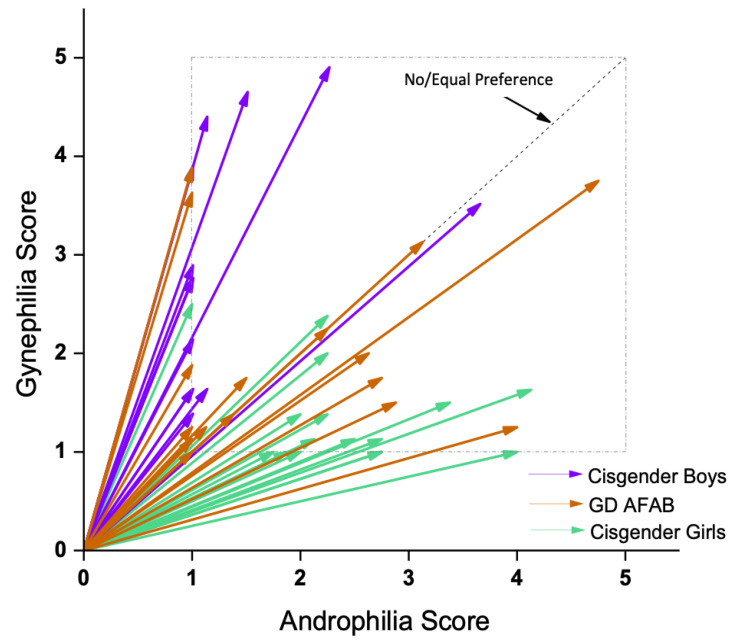
EROS results. Cisgender girls (green) were predominantly androphilic (<45°), cisgender boys (purple) were predominantly gynephilic (>45°) and GD AFAB (adolescents assigned female at birth who experience gender dysphoria) individuals (dark orange) expressed a range of sexual attraction targets.

Regarding sexual orientation, a main effect of group was found for degree of androphilia-gynephilia, whereas strength of attractions showed no significant group differences (Table 1). For degree of androphilia-gynephilia, cisgender boys were more gynephilic than both GD AFAB (MD = 18.21, *SE* = 4.74, *p* < 0.001) and cisgender girls (MD = 33.66, *SE* = 4.67, *p* < 0.001). GD AFAB were more gynephilic than cisgender girls (MD = 15.44, *SE* = 4.51, *p* = 0.001). Examination of the scores showed that cisgender boys tended to be gynephilic, cisgender girls tended to be androphilic, and GD AFAB had a range of sexual attractions with a cluster of individuals in the ambiphilic or asexual range (see Figure 2).

Androphilia scores were not related to gynephilia scores across participants (*r* = −0.09, *p* = 0.570, *n* = 47), within cisgender boys (*r* = 0.43, *p* = 0.129, *n* = 14), within GD AFAB (*r* = 0.25, *p* = 0.360, *n* = 16), or within cisgender girls (*r* = −0.16, *p* = 0.553, *n* = 17). Likewise, strength of attractions was not related to degree of androphilia-gynephilia across participants (*r* = 0.05, *p* = 0.765, *n* = 47), within cisgender boys (*r* = 0.24, *p* = 0.418, *n* = 14), within GD AFAB participants (*r* = −0.15, *p* = 0.570, *n* = 16), or within cisgender girls (*r* = −0.35, *p* = 0.163, *n* = 17). Strength of attractions was significantly correlated with age across all participants (*r* = 0.53, *p* < 0.001, *n* = 47), within cisgender boys (*r* = 0.87, *p* < 0.001, *n* = 14) and cisgender girls (*r* = 0.58, *p* = 0.014, *n* = 17), but not within GD AFAB (*r* = 0.20, *p* = 0.458, *n* = 16). Degree of androphilia-gynephilia was not significantly correlated with age across all participants (*r* = 0.01, *p* = 0.940, *n* = 47), nor within any group: cisgender boys (*r* = 0.39, *p* = 0.168, *n* = 14), GD AFAB (*r* = 0.24, *p* = 0.370, *n* = 16), and cisgender girls (*r* = −0.01, *p* = 0.958, *n* = 17).

### 4.2. Task Partial Least Squares (PLS) Analysis

**Figure 3:**
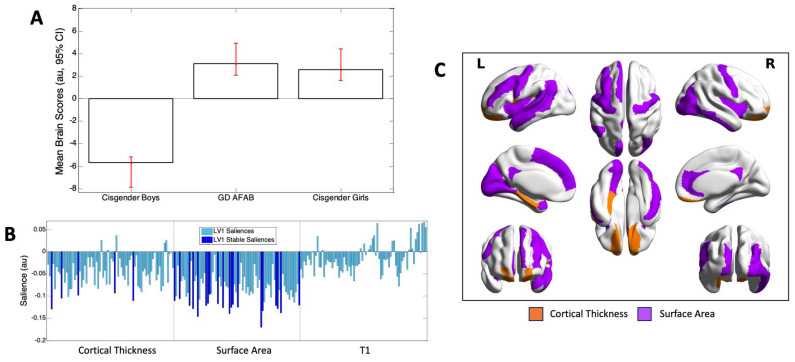
Results of the one significant latent variable from the Task PLS analysis. **Panel A.** Mean brain scores (±95% confidence interval, CI), showing a sex assigned at birth difference on this latent variable (LV): cisgender boys differed from GD AFAB and cisgender girls. Scatterplots of brain scores by design scores for all participants are shown in Figure S2 (Supplementary Materials). **Panel B.** Brain saliences for all regions of interest (ROIs) for cortical thickness, surface area, and T1. The 25 ROIs stably contributing to the sex difference are shown in dark blue and included cortical thickness in 5 regions and surface area in 20 regions. The majority of brain saliences (and all stable saliences) were negative, indicating that cisgender boys generally had thicker cortices and larger surface areas than GD AFAB adolescents and cisgender girls. **Panel C.** Surface projection of stable ROIs: orange = cortical thickness; purple = surface area, created with BrainNet Viewer (version 1.7; [86]). L = left; R = right.

The Task PLS analysis identified one significant latent variable (*p* = 0.000) that accounted for 86.59% of the covariance between group and the ROI data. This latent variable differentiated the cisgender girls and GD AFAB from the cisgender boys. Thus, the overall pattern reflected a sex assigned at birth difference (Figure 3, Panel A). The majority of brain saliences and all stable brain saliences were negative, indicating that cisgender boys had generally thicker cortices and larger surface areas than GD AFAB and cisgender girls. T1 did not reliably contribute to the sex difference. The sex difference was most stably expressed in 25 regions, 5 reflecting cortical thickness and 20 reflecting surface area differences (Figure 3, Panel B). These stable regions were distributed throughout the lobes of the brain, and included primarily frontal, temporal, and occipital ROIs (Table 2; Figure 3, Panel C).

### 4.3. Behavior PLS Analysis

**Figure 4:**
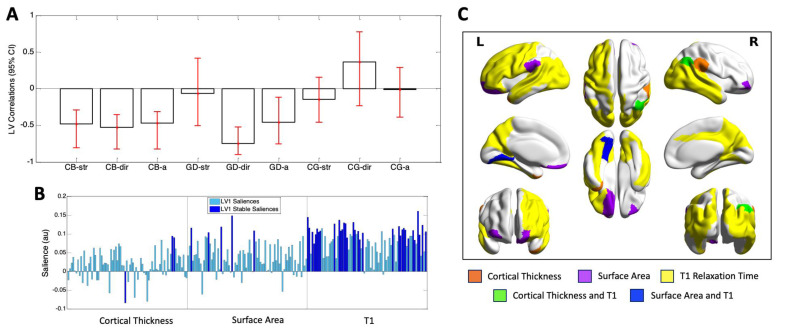
Results of the one significant latent variable from the Behavior PLS analysis. **Panel A.** Correlations of brain scores with behavior (± 95% confidence interval, CI). This latent variable (LV) identified similar and stable correlations in cisgender boys (CB) and GD AFAB (GD), which were stable for age (CB-a, GD-a), degree of androphilia-gynephilia (CB-dir, GD-dir) in both groups, and for strength of sexual attractions in the CB (CB-str). No correlations were stable in the cisgender girls (CG-str, CG-dir, CG-a). Scatterplots of brain scores by behavioral measures for all participants can be found in Figure S3 (Supplementary Materials). **Panel B.** Brain saliences for all ROIs for cortical thickness, surface area, and T1. This latent variable was dominated by stable positive saliences in 33 T1 regions (dark blue). Stable ROIs also included 3 cortical thickness regions and 5 surface area regions. The majority of brain saliences were positive, indicating that in cisgender boys and GD AFAB, shorter T1, thinner (two regions) or thicker cortices (cf. the one region with a stable negative salience), and smaller surface areas correlated with older age and stronger gynephilia. **Panel C.** Surface projection of stable ROIs: orange = cortical thickness only; purple = surface area only; yellow = T1 relaxation time only; green = cortical thickness and T1; blue = surface area and T1, created with BrainNet Viewer (version 1.7; [86]). L = left; R = right.

The Behavior PLS analysis identified one significant latent variable (*p* = 0.000) that accounted for 45.87% of the cross-block covariance between the ROI data and the two EROS variables and age across the groups. This latent variable indicated that cisgender boys and GD AFAB shared similar brain–behavior correlations, with no stable correlations in the cisgender girls. For the cisgender boys, EROS strength of attractions, EROS degree of androphilia-gynephilia, and age were stably related to the ROI data; for the GD AFAB group, similar correlations were found for EROS degree of androphilia-gynephilia and age (Figure 4, Panel A). The majority of brain saliences were positive, indicating that the brain metrics were inversely related to the behavioral measures. The stable regions were dominated by those reflecting correlations of T1 with the behavioral measures, across the brain. Thus, in both cisgender boys and GD AFAB, shorter regional T1s correlated with older age, stronger gynephilia, and—for cisgender boys only—stronger attractions. Across all measures, the brain-behavior correlations in cisgender boys and GD AFAB were most stably expressed in 41 regions, 33 of which reflected regional correlations with T1. The regions contributing to the observed brain-behavior patterns in cortical thickness and surface area regions were fewer, with only 3 stable contributions in cortical thickness regions, and 5 stable contributions in surface area regions (Figure 4, Panel B). With the exception of one cortical thickness region, the brain saliences were positive, reflecting negative correlations with the behavioral measures for both cortical thickness and surface area, similar to what was found for T1. As was found in the Task PLS results, the stable regions were distributed throughout the lobes of the brain (Table 2; Figure 4, Panel C).

## 5. Discussion

Using multivariate PLS analyses, we examined group differences (GD AFAB, cisgender boys, cisgender girls) and associations between sexual attraction, age, and three structural brain metrics: cortical thickness, surface area, and T1 relaxation time. The Task PLS results indicated the structural metrics in GD AFAB adolescents were similar to cisgender girls, and both groups differed from cisgender boys. This sex assigned at birth difference was driven mostly by surface area, with some cortical thickness contributions, such that cisgender boys had larger surface areas and thicker cortices than GD AFAB and cisgender girls. Thus, when examining group differences, a sex difference was revealed, rather than a gender identity difference, which did not support our prediction that the brains of GD AFAB would be similar to their experienced gender. However, our prediction that a sex difference would be driven mostly by surface area was supported.

Many studies, including those with cisgender adolescents, show a sex assigned at birth difference in surface area such that AMAB individuals have larger surface areas than AFAB individuals [13,14,15,36]. Therefore, it is not surprising that surface area contributed the most to the sex difference in the current study. The stable surface area ROIs contributing to this sex difference were distributed across the brain, with most in the frontal, temporal, occipital, and insula/cingulate gyri regions, again aligning with earlier studies of widespread sex differences in surface area [15]. The more limited contribution of cortical thickness to the sex difference was predominant in fronto-temporal ROIs. This aligns with at least one large-scale study that included adolescents and found regions in the frontal and temporal lobes where males had thicker cortices than females, although the regions did not overlap completely with those found in the current study [15]. Not all regions in Wierenga et al. [15] showed thicker cortex in males (vs. females) and there have been mixed results regarding adolescent sex differences in cortical thickness [13,14]. T1 did not stably contribute to the sex difference, consistent with early studies that did not find sex differences in regional T1 [16,17,18].

Our finding that group differences in the Task PLS were related to sex assigned at birth partly aligns with the only other study on the cortical structure of the GD adolescent brain. Hoekzema et al. [5] found that cortical and subcortical gray matter volume in GD AFAB and GD AMAB adolescents not receiving hormone therapy mostly reflected sex assigned at birth and not gender identity, with a few exceptions. Specifically, in a small number of regions displaying sex differences, gender identity differences were also found: in GD AFAB, a smaller left superior medial frontal cortex volume vs. cisgender girls, and in GD AMAB, a smaller volume in the left superior posterior hemisphere of the cerebellum and marginally smaller hypothalamic volumes vs. cisgender boys. We did not find any exceptions to our pattern of sex differences in the Task PLS and in that regard, our study does not fully align with Hoekzema et al. [5].

Some insights regarding mechanisms underlying the development of surface area and cortical thickness have been advanced. The radial unit hypothesis partly explains the prenatal cellular contributions to regional variations in surface area and cortical thickness [87,88]. Specifically, surface area is represented by the number of neuronal columns in an area, determined during in utero brain development by neurons migrating along radial glial cells from the ventricular zone to the cortical plate. Cortical thickness is determined by the number of neurons within these columns. There is evidence from imaging studies that somatic and maturational variables (i.e., age, sex, and physical growth) are more strongly associated with surface area and cortical thickness in the adolescent brain than variables reflecting relatively more environmental influences (e.g., general intelligence, prenatal parental smoking; [89]). If so, it might explain why group differences in the present study were reflected in birth-assigned sex differences in cortical structure, particularly surface area. Imaging studies have further demonstrated that surface area is influenced more by early prenatal environment factors than is cortical thickness [40,41]. The exact mechanisms have not been elucidated, but given the likely prenatal basis for the development of cortical thickness, and particularly surface area, prenatal organizational mechanisms may play a role.

The neurohormonal hypothesis argues that prenatal androgens shape the brain to be organized in a particular manner, including organization of sex differences in the brain. Then, during adolescence, the pubertal surge in sex hormones further influences brain regions to be expressed in a relatively male- or female-typical manner, based on the earlier brain organization [12]. Our finding of a sex assigned at birth difference within the cisgender adolescents aligns with the neurohormonal hypothesis. Thus, variation in prenatal androgen exposure could be one mechanism that produces larger surface areas and somewhat thicker cortices in individual AMAB versus AFAB. However, despite a likely prenatal basis for the development of these two structural brain features, they continue to develop throughout childhood and adolescence [13,35,36,37]. Thus, additional mechanisms, which are not necessarily mutually exclusive, may play a role. For example, circulating pubertal hormones could influence neural reorganization [90]. The pubertal increase in steroid sex hormones and accompanying physical maturation have been related to cortical development in cisgender adolescents [91]. Furthermore, the effects of these physical changes on the brain are speculated to be influenced further by indirect mechanisms such as interactions with the social environment [92].

In contrast to the group difference associated with sex assigned at birth, when brain structure was assessed in relation to sexual orientation and age, the findings indicated similarities between GD AFAB and cisgender boys that were not shared with cisgender girls. In the Behavior PLS analysis, we found that cisgender boys and GD AFAB adolescents had similar correlations between brain measures and age and sexual orientation. Specifically, gynephilia, older age, and stronger attractions (in cisgender boys only) were related predominantly to shorter T1 in regions distributed throughout the frontal, temporal, parietal, and occipital lobes. Thus, in GD AFAB and cisgender boys, brain patterns showed similarities in how they varied in relation to sexual orientation as well as developmentally (i.e., in relation to age). Given these patterns were not seen in cisgender girls, the similarities between GD AFAB and cisgender boys in the context of sexual attractions and age provide some support for our prediction that the brains of GD AFAB would be similar to their experienced gender.

For T1, 33 regions stably contributed to the similarities between GD AFAB and cisgender boys such that T1 was shorter in older GD AFAB and cisgender boys. Shorter T1s are associated with more myelination and other macromolecules, more iron, less free water, and increased gray matter tissue density [43]. T1 typically shortens in gray matter with age, including during adolescence [17,18,43,44,45,46]. The adolescent brain also experiences changes in surface area and cortical thickness, with some minor growth in surface area and decreases in cortical thickness to allow for the increase in white matter (without much growth in brain volume) during this time [13,35,36,37,42]. In our sample, only five surface area regions and three cortical thickness regions combined with the globally shorter T1s to contribute to the observed similarities in correlations between brain metrics and sexual orientation measures and age in GD AFAB and cisgender boys. Thus, one way in which the adolescent brains of GD AFAB are similar to those of cisgender boys could be through a similarity in timing of age-related changes in mostly cortical tissue microstructure related to sexual attraction.

The results of the Behavior PLS suggest that only focusing on cortical thickness and surface area may overlook insights provided by mechanisms that influence variation in cortical tissue microstructure. These mechanisms are not fully understood, but findings related to the development of dendritic arborization and myelin could provide insights (e.g., [93]). Given associations of the brain metrics with sexual orientation and age, mechanisms that influence these variables may also provide a good starting point for speculation. There is some support for the neurohormonal hypothesis in underlying the development of sexual orientation, particularly in same-sex attracted cisgender women (i.e., gynephilia) [23,30,94]. Our results show that cortical structure and microstructure of GD AFAB is similar to cisgender boys in the context of gynephilia, which is consistent with the neurohormonal hypothesis and the role of prenatal androgens. However, alternative explanations are also plausible. For instance, if the cortical microstructure similarities are attributable to myelination [95], it has been demonstrated in rodent models that estradiol decreases myelination during puberty [96], whereas androgen exposure leads to thicker myelin sheaths [97]. However, these cellular properties have not yet been related to any psychological traits in humans (e.g., gynephilia).

Shorter T1 was related to stronger attractions in cisgender boys only, highlighting the likely developmental importance of this aspect of sexual attractions. Development of the strength of attractions, and mechanisms underlying the development of this aspect of sexual orientation, are understudied in the sexual orientation literature. Xu et al. [34] recently found that the intensity of sexual behaviors increased (i.e., from hugging to having sexual intercourse) with age in cisgender adolescents, and the likelihood of engagement in same-sex behaviors increased in later adolescence, but these relationships were found in both cisgender boys and cisgender girls, strength of sexual attractions was not examined, and the findings were not discussed further. Given that cisgender boys showed strong relationships between tissue microstructure, degree of androphilia-gynephilia, strength of sexual attractions, and age, incorporating similar measures in future studies of gender and sexual orientation in adolescence is warranted.

### Limitations

The present results should be interpreted with caution in light of criticisms of neuroimaging research conducted with small sample sizes [98]. We ameliorated some concerns of our small sample size by utilizing a multivariate statistical approach and by interpreting distributed patterns rather than focusing on individual brain regions. Furthermore, our results largely align with the only other study of adolescent GD brain structure, which had a similar sample size of GD AFAB adolescents not on puberty blockers or gender-affirming hormone therapy, but did have larger samples of cisgender adolescents [5].

An additional limitation is that the cross-sectional non-experimental design does not allow us to determine causal relationships. With our study design, we cannot infer whether changes in the measured brain features result in GD and/or sexual orientation, or vice versa. Moreover, we cannot know whether changes in the measured brain features and GD and/or sexual orientation are the result of a third variable (e.g., a shared underlying cause such as variation in prenatal hormone exposure). We also used a convenience sample, which may affect representativeness. We did not directly examine other non-brain mechanisms (e.g., peripheral hormone levels) to interpret the results. Finally, we did not measure consumption of various substances (e.g., water, caffeine, smoking) prior to the MRI scan, or quantify differences in vasculature and metabolism (e.g., cerebral blood flow) that have been shown to impact measurements of T1 and cortical thickness (e.g., [99,100,101]).

Our results can only address GD AFAB adolescents given GD AMAB adolescents were not included. Also, our results cannot generalize to the broader sexual orientation literature given the small number of cisgender same-sex attracted adolescents in this sample. The restricted range in degree of androphilia-gynephilia in cisgender boys and cisgender girls may have also impacted our findings in the Behavior PLS. Furthermore, our results cannot generalize to ages beyond 12- to 17-years-old. Studies on adults have examined cortical structure related to GD [9,26,102,103,104,105,106,107,108], but we know of no studies examining cortical structure of GD children. Lastly, we examined three cortical structural brain features; other aspects of adolescent GD brain structure (e.g., white matter microstructure) await investigation. 

## 6. Conclusions

In our sample of GD AFAB adolescents not receiving hormone therapy, cisgender girls, and cisgender boys, a multivariate sex assigned at birth difference in cortical structure was identified, driven mostly by variability in surface area. When assessing correlations of cortical structure with sexual orientation and age, the brains of GD AFAB adolescents were similar to cisgender boys, driven mostly by T1 relaxation time. Our findings suggest that the cortical structure of GD AFAB adolescents aligns with the experienced gender in the context of age-related changes in sexual attractions during adolescence reflected mostly in tissue microstructure.

## Figures and Tables

**Table 1:** Descriptive statistics for demographic and psychosexual variables.

	Cisgender Boys	GD AFAB	Cisgender Girls	*F* (*df*)	*p*
*n*	14	16	17		
Age (months)					
*M*	184.93	193.38	191.71	0.75 (2, 44)	0.480
*SD*	25.61	14.20	19.00		
Range	147–216	162–216	152–214		
GIDYQ-AA					
*M*	4.91	2.21	4.90	693.34 (2, 44) ^c^	<0.001
*SD*	0.12	0.35	0.15		
Range (1–5) ^a^	4.63–5.00	1.74–3.04	4.48–5.00		
Strength of attractions					
*M*	3.25	3.00	2.83	0.54 (2, 44) ^d^	0.587
*SD*	1.28	1.30	0.76		
Range (1.41–7.07) ^b^	1.70–5.37	1.41–6.05	1.41–4.44		
Degree of androphilia-gynephilia					
*M*	64.60	46.38	30.94	25.93 (2, 44)	<0.001
*SD*	9.29	15.23	13.18		
Range (11–79) ^b^	43.96–75.55	17.35–75.55	14.04–68.20		

Note. GIDYQ-AA = Gender Identity/Gender Dysphoria Questionnaire for Adolescents and Adults; GD = gender dysphoria; AFAB = assigned female at birth. ^a^ Absolute range. ^b^ Possible range for magnitude and phase (θ) based on EROS scores with absolute range of 1 to 5. ^c^ A significant Levene’s test (*p* = 0.009) warrants reporting of the robust tests of equality of means via the Welch test statistic (2, 27.46) = 422.50, *p* < 0.001, and the Brown–Forsythe test statistic (2, 24.31) = 713.80, *p* < 0.001. ^d^ A significant Levene’s test (*p* = 0.032) warrants reporting of the robust tests of equality of means via the Welch test statistic (2, 25.76) = 0.60, *p* = 0.557, and the Brown–Forsythe test statistic (2, 35.89) = 0.52, *p* = 0.599.

**Table 2:** Stable ROIs contributing to group differences from Task PLS and Behavior PLS.

Task PLS			Behavior PLS	
SA/CT	Hem.	ROI	SA/CT/T1	Hem.
Frontal Lobe				
CT	Left	Superior Frontal Gyrus: Orbital Part	SA	Left
CT	Right	Superior Frontal Gyrus: Orbital Part		
SA	Left	Middle Frontal Gyrus	T1	Left
CT	Left	Rolandic Operculum	T1	Left
SA	Left	Superior Frontal Gyrus: Medial		
SA	Left	Precentral Gyrus	T1	Left
SA	Right	Precentral Gyrus		
CT	Right	Gyrus Rectus		
		Gyrus Rectus	SA	Left
SA	Left	Supplementary Motor Area		
		Middle Frontal Gyrus: Orbital Part	T1	Left
		Middle Frontal Gyrus: Orbital Part	SA	Right
		Superior Frontal Gyrus: Dorsolateral	T1	Left
		Inferior Frontal Gyrus: Opercular Part	T1	Left
		Inferior Frontal Gyrus: Triangular Part	T1	Left
		Inferior Frontal Gyrus: Orbital Part	T1	Left
Parietal Lobe				
SA	Left	Angular Gyrus	T1	Left
		Angular Gyrus	CT; T1	Right
		Supramarginal Gyrus	SA	Left
		Supramarginal Gyrus	CT	Right
		Postcentral Gyrus	T1	Left
		Precuneus	T1	Left
		Precuneus	T1	Right
Temporal Lobe				
CT	Left	Parahippocampal Gyrus		
SA	Left	Temporal Pole: Superior Temporal Gyrus		
SA	Left	Superior Temporal Gyrus	T1	Left
		Superior Temporal Gyrus	T1	Right
SA	Left	Middle Temporal Gyrus	T1	Left
		Middle Temporal Gyrus	T1	Right
SA	Right	Inferior Temporal Gyrus	T1	Right
		Temporal Pole: Middle Temporal Gyrus	CT	Left
		Fusiform Gyrus	T1	Left
		Heschl Gyrus	T1	Left
		Heschl Gyrus	T1	Right
Occipital Lobe				
		Middle Occipital Gyrus	T1	Left
SA	Right	Middle Occipital Gyrus	T1	Right
		Inferior Occipital Gyrus	T1	Left
SA	Right	Inferior Occipital Gyrus	T1	Right
SA	Left	Lingual Gyrus	SA; T1	Left
		Lingual Gyrus	T1	Right
SA	Left	Calcarine Fissure and Surrounding Cortex		
SA	Left	Cuneus	T1	Left
		Cuneus	T1	Right
		Superior Occipital Gyrus	T1	Left
		Superior Occipital Gyrus	T1	Left
Insula and Cingulate Gyri				
SA	Left	Insula		
SA	Right	Insula		
SA	Right	Anterior Cingulate and Paracingulate Gyri		
SA	Left	Posterior Cingulate Gyrus		
SA	Right	Posterior Cingulate Gyrus	T1	Right
		Median Cingulate and Paracingulate Gyri	T1	Right

Note. PLS = partial least squares, SA = surface area, CT = cortical thickness, T1 = T1 relaxation time, Hem. = hemisphere, ROI = region of interest.

## Data Availability

Most code and all of the numerical data presented in this study are openly available in Scholar’s Portal Dataverse at doi:10.5683/SP2/B3HMPQ [47]. Computations were partly performed on the Niagara supercomputer at the SciNet HPC Consortium. SciNet is funded by: the Canada Foundation for Innovation; the Government of Ontario; Ontario Research Fund—Research Excellence; and the University of Toronto. minc-bpipe-library is available at https://github.com/CobraLab/minc-bpipe-library.git; FSL tool library is available at https://fsl.fmrib.ox.ac.uk/fsl/fslwiki/; PLS software is available at https://www.rotman-baycrest.on.ca/index.php?section=84; BrainNet Viewer is available at https://www.nitrc.org/projects/bnv/.

## Supplementary Materials

The following are available online at https://www.mdpi.com/2077-0383/10/2/345/s1, Figure S1: Examples of T1 histograms, Figure S2: Task PLS results (brain scores by design scores), Figure S3: Scatterplots showing the brain score-behavior correlations for all groups and behavioral measures, Table S1: Descriptive statistics for additional demographic variables, Table S2: Summary of correlation analysis between demographic variables and ROI data across participants.

## References

[1] American Psychiatric Association (2013). Diagnostic and Statistical Manual of Mental Disorders.

[2] Aitken M., Steensma T.D., Blanchard R., VanderLaan D.P., Wood H., Fuentes A., Spegg C., Wasserman L., Ames M., Fitzsimmons C.L. (2015). Evidence for an altered sex ratio in clinic-referred adolescents with gender dysphoria. J. Sex. Med..

[3] De Graaf N.M., Giovanardi G., Zitz C., Carmichael P. (2018). Sex ratio in children and adolescents referred to the Gender Identity Development Service in the UK (2009–2016) [Letter to the Editor]. Arch. Sex. Behav..

[4] Meerwijk E.L., Sevelius J.M. (2017). Transgender population size in the United States: A meta-regression of population-based probability samples. Am. J. Pub. Health.

[5] Hoekzema E., Schagen S.E.E., Kreukels B.P.C., Veltman D.J., Cohen-Kettenis P.T., Delemarre-van de Waal H., Bakker J. (2015). Regional volumes and spatial volumetric distribution of gray matter in the gender dysphoric brain. Psychoneuroendocrinology.

[6] Guillamon A., Junque C., Gomez-Gil E. (2016). A review of the status of brain structure research in transsexualism. Arch. Sex. Behav..

[7] Hines M. (2020). Neuroscience and sex/gender: Looking back and forward. J. Neurosci..

[8] Meyer-Bahlburg H.F.L. (2011). Transsexualism (“gender identity disorder”)—A CNS-limited form of intersexuality?. Adv. Exp. Med. Biol..

[9] Nguyen H.B., Loughead J., Lipner E., Hantsoo L., Kornfield S.L., Epperson C.N. (2018). What has sex got to do with it? The role of hormones in the transgender brain. Neuropsychopharmacology.

[10] Ristori J., Cocchetti C., Romani A., Mazzoli F., Vignozzi L., Maggi M., Fisher A.D. (2020). Brain sex differences related to gender identity development: Genes or hormones?. Int. J. Mol. Sci..

[11] Roselli C.E. (2018). Neurobiology of gender identity and sexual orientation. J. Neuroendocrinol..

[12] Hines M. (2011). Prenatal endocrine influences on sexual orientation and on sexually differentiated childhood behavior. Front. Neuroendocrinol..

[13] Raznahan A., Shaw P., Lalonde F., Stockman M., Wallace G.L., Greenstein D., Clasen L., Gogtay N., Giedd J.N. (2011). How does your cortex grow?. J. Neurosci..

[14] Wierenga L.M., Langen M., Oranje B., Durston S. (2014). Unique developmental trajectories of cortical thickness and surface area. NeuroImage.

[15] Wierenga L.M., Doucet G.E., Dima D., Agartz I., Aghajani M., Akudjedu T.N., Albajes-Eizagirre A., Alnaes D., Alpert K.I., Andreassen O.E. (2020). Greater male than female variability in regional brain structure across the lifespan. Hum. Brain Mapp..

[16] Breger R.K., Yetkin Z., Fischer M.E., Papke R.A., Haughton V.M., Rimm A.A. (1991). T1 and T2 in the cerebrum: Correlation with age, gender, and demographic factors. Radiology.

[17] Cho S., Jones D., Reddick W.E., Ogg R.J., Steen R.G. (1997). Establishing norms for age-related changes in proton T1 of human brain tissue in vivo. Magn. Reson. Imaging.

[18] Steen R.G., Ogg R.J., Reddick W.E., Kingsley P.B. (1997). Age-related changes in the pediatric brain: Quantitative MR evidence of maturational changes during adolescence. Am. J. Neuroradiol..

[19] Gennatas E.D., Avants B.B., Wolf D.H., Satterthwaite T.D., Ruparel K., Ciric R., Hakonarson H., Gur R.E., Gur R.C. (2017). Age-related effects and sex differences in gray matter density, volume, mass, and cortical thickness from childhood to young adulthood. J. Neurosci..

[20] Rabinowicz T., Petetot J.M.-C., Khoury J.C., de Courten-Myers G.M. (2009). Neocortical maturation during adolescence: Change in neuronal soma dimension. Brain Cogn..

[21] Bonifacio J.H., Maser C., Stadelman K., Palmert M. (2019). Management of gender dysphoria in adolescents in primary care. Can. Med. Assoc. J..

[22] Balthazart J. (2020). Sexual partner preference in animals and humans. Neurosci. Biobehav. Rev..

[23] Bogaert A.F., Skorska M.N. (2020). A short review of biological research on the development of sexual orientation. Horm. Behav..

[24] Ellis L., Ames M.A. (1987). Neurohormonal functioning and sexual orientation: A theory of homosexuality-heterosexuality. Psychol. Bull..

[25] Hines M. (2011). Gender development and the human brain. Annu. Rev. Neurosci..

[26] Manzouri A., Savic I. (2019). Possible neurobiological underpinnings of homosexuality and gender dysphoria. Cereb. Cortex.

[27] Calzo J.P., Blashill A.J. (2018). Child sexual orientation and gender identity in the Adolescent Brain Cognitive Development Cohort Study. JAMA Pediatr..

[28] Herdt G., McClintock M.K. (2000). The magical age of 10. Arch. Sex. Behav..

[29] McClintock M.K., Herdt G. (1996). Rethinking puberty: The development of sexual attraction. Curr. Dir. Psychol. Sci..

[30] Bailey J.M., Vasey P.L., Diamond L.M., Breedlove S.M., Vilain E., Epprecht M. (2016). Sexual orientation, controversy, and science. Psychol. Sci. Public Interest.

[31] Cavazos-Rehg P.A., Krauss M.J., Spitznagel E.L., Schootman M., Bucholz K.K., Peipert J.F., Sanders-Thompson V., Cottler L.B., Bierut L.J. (2009). Age of sexual debut among US adolescents. Contraception.

[32] Savin-Williams R.C., Ream G.L. (2007). Prevalence and stability of sexual orientation components during adolescence and young adulthood. Arch. Sex. Behav..

[33] Savin-Williams R.C., Joyner K., Rieger G. (2012). Prevalence and stability of self-reported sexual orientation identity during young adulthood. Arch. Sex. Behav..

[34] Xu Y., Norton S., Rahman Q. (2020). Adolescent sexual behavior patterns in a British birth cohort: A latent class analysis. Arch. Sex. Behav..

[35] Gilmore J.H., Knickmeyer R.C., Gao W. (2018). Imaging structural and functional brain development in early childhood. Nat. Rev. Neurosci..

[36] Kaczkurkin A.N., Raznahan A., Satterthwaite T.D. (2019). Sex differences in the developing brain: Insights from multimodal neuroimaging. Neuropsychopharmacol..

[37] Lyall A.E., Shi F., Geng X., Woolson S., Li G., Wang L., Hamer R.M., Shen D., Gilmore J.H. (2015). Dynamic development of regional cortical thickness and surface area in early childhood. Cereb. Cortex.

[38] Panizzon M.S., Fennema-Notestine C., Eyler L.T., Jernigan T.L., Prom-Wormley E., Neale M., Jacobson K., Lyons M.J., Grant M.D., Franz C.E. (2009). Distinct genetic influences on cortical surface area and cortical thickness. Cereb. Cortex.

[39] Winkler A.M., Kochunov P., Blangero J., Almasy L., Zilles K., Fox P.T., Duggirala R., Glahn D.C. (2010). Cortical thickness or grey matter volume? The importance of selecting the phenotype for imaging genetics studies. NeuroImage.

[40] Jha S.C., Xia K., Ahn M., Girault J.B., Li G., Wang L., Shen D., Zou F., Zhu H., Styner M. (2019). Environmental influences on infant cortical thickness and surface area. Cereb. Cortex.

[41] Raznahan A., Greenstein D., Lee N.R., Clasen L.S., Giedd J.N. (2012). Prenatal growth in humans and postnatal brain maturation into late adolescence. Proc. Natl. Acad. Sci. USA.

[42] Colby J.B., O’Hare E.D., Bramen J.E., Sowell E.R., Rubenstein J.L.R., Rakic P. (2013). Structural brain development: Birth through adolescence. Neural Circuit Development and Function in the Brain.

[43] Deoni S.C.L. (2010). Quantitative relaxometry of the brain. Top. Magn. Reson. Imaging.

[44] Gracien R.-M., Nurnberger L., Hok P., Hof S.-M., Reitz S.C., Rub U., Steinmetz H., Hilker-Roggendorf R., Klein J.C., Deichmann R. (2017). Evaluation of brain ageing: A quantitative longitudinal MRI study over 7 years. Eur. Radiol..

[45] Saito N., Sakai O., Ozonoff A., Jara H. (2009). Relaxo-volumetric multispectral quantitative magnetic resonance imaging of the brain over the human lifespan: Global and regional aging patterns. Magn. Reson. Imaging.

[46] Seiler A., Schongrundner S., Stock B., Noth U., Hattingen E., Steinmetz H., Klein J.C., Baudrexel S., Wagner M., Deichmann R. (2020). Cortical aging—New insights with multiparametric quantitative MRI. Aging.

[47] Skorska M.N., Chavez S., Devenyi G.A., Patel R., Thurston L.T., Lai M.-C., Zucker K.J., Chakravarty M.M., Lobaugh N.J., VanderLaan D.P. (2020). Data from: A multi-modal MRI analysis of cortical structure in relation to gender dysphoria, sexual orientation, and age in adolescents. Sch. Portal Dataverse.

[48] Deogracias J.J., Johnson L.L., Meyer-Bahlburg H.F.L., Kessler S.J., Schober J.M., Zucker K.J. (2007). The Gender Identity/Gender Dysphoria Questionnaire for Adolescents and Adults. J. Sex. Res..

[49] Singh D., Deogracias J.J., Johnson L.J., Bradley S.J., Kibblewhite S.J., Owen-Anderson A., Badali-Peterson M., Meyer-Bahlburg H.F.L., Zucker K.J. (2010). The Gender Identity/Gender Dysphoria Questionnaire for Adolescents and Adults: Further validity evidence. J. Sex. Res..

[50] Zucker K.J., Bradley S.J., Owen-Anderson A., Kibblewhite S.J., Wood H., Singh D., Choi K. (2012). Demographics, behavior problems, and psychosexual characteristics of adolescents with gender identity disorder or transvestic fetishism. J. Sex. Marital Ther..

[51] Drummond K.D., Bradley S.J., Badali-Peterson M., Zucker K.J. (2008). A follow-up study of girls with gender identity disorder. Dev. Psychol..

[52] Storms M.D. (1980). Theories of sexual orientation. J. Pers. Soc. Psychol..

[53] Zucker K.J., Bradley S.J., Oliver G., Blake J., Fleming S., Hood J. (1996). Psychosexual development of women with congenital adrenal hyperplasia. Horm. Behav..

[54] Deoni S.C.L. (2007). High-resolution T1 mapping of the brain at 3T with driven equilibrium single pulse observation of T1 with high-speed incorporation of RF field inhomogeneities (DESPOT1-HIFI). J. Magn. Reson. Imaging.

[55] Wang J., Qiu M., Kim H., Constable R.T. (2006). T1 Measurements incorporating flip angle calibration and correction in vivo. J. Mag. Reson..

[56] Chavez S., Stanisz G.J. (2012). A novel method for simultaneous 3D B1 and T1 mapping: The method of slopes (MoS). NMR Biomed..

[57] Loken C., Gruner D., Groer L., Peltier R., Bunn N., Craig M., Henriques T., Dempsey J., Yu C.-H., Chen J. (2010). SciNet: Lessons learned from building a power-efficient top-20 system and data centre. J. Phys. Conf. Ser..

[58] Ponce M., van Zon R., Northrup S., Gruner D., Chen J., Ertinaz F., Fedoseev A., Groer L., Mao F., Mundim B.C. Deploying a top-100 supercomputer for large parallel workloads: The Niagara Supercomputer. Proceedings of the Practice and Experience in Advanced Research Computing (PEARC 2019).

[59] Tustison N.J., Avants B.B., Cook P.A., Zheng Y., Egan A., Yushkevich P.A., Gee J.C. (2010). N4ITK: Improved N3 bias correction. IEEE Trans. Med. Imaging.

[60] Eskildsen S.F., Coupe P., Fonov V., Manjon J.V., Leung K.K., Guizard N., Wassef S.H., Ostergaard L.R., Collins D.L. (2012). The Alzheimer’s Neuroimaging Initiative. BEaST: Brain extraction based on nonlocal segmentation technique. NeuroImage.

[61] Bedford S.A., Park M.T.M., Devenyi G.A., Tullo S., Germann J., Patel R., Anagnostou E., Baron-Cohen S., Bullmore E.T., Chura L.R. (2020). Large-scale analyses of the relationship between sex, age and intelligence quotient heterogeneity and cortical morphometry in autism spectrum disorder. Mol. Psychiatry.

[62] Patel S., Patel R., Park M.T.M., Masellis M., Knight J., Chakravarty M.M. (2018). Heritability estimates of cortical anatomy: The influence and reliability of different estimation strategies. NeuroImage.

[63] Ad-Dab’bagh Y., Einarson D., Lyttelton O., Muehlboeck J.-S., Mok K., Ivanov O., Vincent R.D., Lepage C., Lerch J., Fombonne E. The CIVET image-processing environment: A fully automated comprehensive pipeline for anatomical neuroimaging research. Proceedings of the 12th Annual Meeting of the Organization for Human Brain Mapping.

[64] Zijdenbos A.P., Forghani R., Evans A.C. (2002). Automatic “pipeline” analysis of 3-D MRI data for clinical trials: Application to multiple sclerosis. IEEE Trans. Med. Imaging.

[65] Lerch J.P., Evans A.C. (2005). Cortical thickness analysis examined through power analysis and a population simulation. NeuroImage.

[66] Lyttelton O., Boucher M., Robbins S., Evans A.C. (2007). An unbiased iterative group registration template for cortical surface analysis. NeuroImage.

[67] Tzourio-Mazoyer N., Landeau B., Papathanassiou D., Crivello F., Etard O., Delcroix N., Mazoyer B., Joliot M. (2002). Automated anatomical labeling of activations in SPM using a macroscopic anatomical parcellation of the MNI MRI single-subject brain. NeuroImage.

[68] Chavez S. Calibrating variable flip angle (VFA)-based T1 maps: When and why a simple scaling factor is justified. Proceedings of the International Society of Magnetic Resonance in Medicine (ISMRM).

[69] Avants B.B., Tustison N.J., Song G., Cook P.A., Klein A., Gee J.C. (2011). A reproducible evaluation of ANTs similarity metric performance in brain image registration. NeuroImage.

[70] Tustison N.J., Avants B.B. (2013). Explicit B-spline regularization in diffeomorphic image registration. Front. Neuroinform..

[71] (2017). MATLAB.

[72] Wechsler D. (1991). Wechsler Intelligence Scale for Children.

[73] Wechsler D. (2008). Wechsler Adult Intelligence Scale.

[74] Wechsler D. (2014). Wechsler Intelligence Scale for Children.

[75] Petersen A.C., Crockett L., Richards M., Boxer A. (1988). A self-report measure of pubertal status: Reliability, validity, and initial norms. J. Youth Adolesc..

[76] Achenbach T.M. (1991). Manual for the Youth Self-Report and 1991 Profile.

[77] Achenbach T.M., Edelbrock C. (1986). Manual for the Youth Self-Report and Profile.

[78] Achenbach T.M., Rescorla L.A. (2001). Manual for the ASEBA School-Age Forms & Profiles.

[79] Ruigrok A.N.V., Salimi-Khorshidi G., Lai M.-C., Baron-Cohen S., Lombardo M.V., Tait R.J., Suckling J. (2014). A meta-analysis of sex differences in human brain structure. Neurosci. Biobehav. Rev..

[80] Ducharme S., Albaugh M.D., Nguyen T.-V., Hudziak J.J., Mateos-Perez J.M., Labbe A., Evans A.C., Karama S. (2016). The Brain Development Cooperative Group. Trajectories of cortical thickness maturation in normal brain development—The importance of quality control procedures. NeuroImage.

[81] McIntosh A.R., Bookstein F.L., Haxby V., Grady C.L. (1996). Spatial pattern analysis of functional brain images using partial least squares. NeuroImage.

[82] McIntosh A.R., Lobaugh N.J. (2004). Partial least squares analysis of neuroimaging data: Applications and advances. NeuroImage.

[83] Chen X.J., Kovacevic N., Lobaugh N.J., Sled J.G., Henkelman R.M., Henderson J.T. (2005). Neuroanatomical differences between mouse strains as shown by high-resolution 3D MRI. NeuroImage.

[84] Geladi P., Kowalski B.R. (1986). Partial Least-Squares Regression: A Tutorial. Anal. Chim. Acta.

[85] (2014). MATLAB.

[86] Xia M., Wang J., He Y. (2013). BrainNet Viewer: A network visualization tool for human brain connectomics. PLoS ONE.

[87] Rakic P. (1988). Specification of cerebral cortical areas. Science.

[88] Rakic P. (2007). The radial edifice of cortical architecture: From neuronal silhouettes to genetic engineering. Brain Res. Rev..

[89] Modabbernia A., Reichenberg A., Ing A., Moser D.A., Doucet G.E., Artiges E., Banaschewski T., Barker G.J., Becker A., Bokde A.L.W. (2020). Linked patterns of biological and environmental covariation with brain structure in adolescence: A population-based longitudinal study. Mol. Psychiatry.

[90] Schulz K.M., Molenda-Figueira H.A., Sisk C.L. (2009). Back to the future: The organizational-activational hypothesis adapted to puberty and adolescence. Horm. Behav..

[91] Herting M.M., Sowell E.R. (2017). Puberty and structural brain development in humans. Front. Neuroendocrinol..

[92] Berenbaum S.A., Beltz A.M., Corely R. (2015). The importance of puberty for adolescent development: Conceptualization and measurement. Adv. Child. Dev. Behav..

[93] Cafiero R., Brauer J., Anwander A., Friederici A.D. (2019). The concurrence of cortical surface area expansion and white matter myelination in human brain development. Cereb. Cortex.

[94] Breedlove S.M. (2017). Prenatal influences on human sexual orientation: Expectations versus data. Arch. Sex. Behav..

[95] Stuber C., Morawski M., Schafer A., Labadie C., Wahnert M., Leuze C., Streigher M., Barapatre N., Reimann K., Geyer S. (2014). Myelin and iron concentration in the human brain: A quantitative study of MRI contrast. NeuroImage.

[96] Juraska J.M., Markham J.A. (2004). The cellular basis for volume changes in the rat cortex during puberty: White and gray matter. Ann. N. Y. Acad. Sci..

[97] Abi Ghanem C., Degerny C., Hussain R., Liere P., Pianos A., Tourpin S., Habert R., Macklin W.B., Schumacher M., Ghoumari A.M. (2017). Long-lasting masculinizing effects of postnatal androgens on myelin governed by the brain androgen receptor. PLoS Genet..

[98] Button K.S., Ioannidis J.P.A., Mokrysz C., Nosek B.A., Flint J., Robinson E.S.J., Munafo M.R. (2013). Power failure: Why small sample size undermines the reliability of neuroscience. Nat. Rev. Neurosci..

[99] Akkermans S.E.A., van Rooij D., Rommelse N., Hartman C.A., Hoekstra P.J., Franke B., Mennes M., Buitelaar J.K. (2017). Effect of tobacco smoking on frontal cortical thickness development: A longitudinal study in a mixed cohort of ADHD-affected and -unaffected youth. Eur. Neuropsychopharmacol..

[100] Ge Q., Peng W., Zhang J., Weng X., Zhang Y., Liu T., Zang Y.-F., Wang Z. (2017). Short-term apparent brain tissue changes are contributed by cerebral blood flow alterations. PLoS ONE.

[101] Tardif C.L., Steele C.J., Lampe L., Bazin P.-L., Ragert P., Villringer A., Gauthier C.J. (2017). Investigation of the confounding effects of vasculature and metabolism on computational anatomy studies. NeuroImage.

[102] Baldinger-Melich P., Castro M.F.U., Seiger R., Ruef A., Dwyer D.B., Kranz G.S., Klobl M., Kambeitz J., Kaufmann U., Windischberger C. (2019). Sex matters: A multivariate pattern analysis of sex- and gender-related neuroanatomical differences in cis- and transgender individuals using structural magnetic resonance imaging. Cereb. Cortex.

[103] Burke S.M., Manzouri A.H., Dhejne C., Bergstrom K., Arver S., Feusner J.D., Savic-Berglund I. (2018). Testosterone effects on the brain in transgender men. Cereb. Cortex.

[104] Flint C., Forster K., Koser S.A., Konrad C., Zwitserlood P., Berger K., Hermesdorf M., Kircher T., Nenadic I., Krug A. (2020). Biological sex classification with structural MRI data shows increased misclassification in transgender women. Neuropsychopharmacology.

[105] Kilpatrick L.A., Holmberg M., Manzouri A., Savic I. (2019). Cross sex hormone treatment is linked with a reversal of cerebral patterns associated with gender dysphoria to the baseline of cisgender controls. Eur. J. Neurosci..

[106] Kim G.-W., Kim Y.-H., Park K., Jeong G.-W. (2019). A comparative study of white matter volume between postoperative female-to-male transsexuals and healthy female. Int. J. Impot. Res..

[107] Schneider M.A., Spritzer P.M., Suh J.S., Minuzzi L., Frey B.N., Schwarz K., Costa A.B., da Silva D.C., Garcia C.C.G., Fontanari A.M.V. (2020). The link between estradiol and neuroplasticity in transgender women after gender-affirming surgery: A bimodal hypothesis. Neuroendocrinology.

[108] Starcevic A., Dakovic M., Radojicic Z., Filipovic B. (2020). A structural magnetic resonance imaging study in therapy naïve transsexual individuals. Folia Morphol..

