# Supplementary material for: A Multi-Modal MRI Analysis of Cortical Structure in Relation to Gender Dysphoria, Sexual Orientation, and Age in Adolescents
Source: J Clin Med. 2021 Jan 18;10(2):345. doi: 10.3390/jcm10020345 (PMC7831120; doi:10.3390/jcm10020345)
Supplement: Supplementary file 1 [file jcm-10-00345-s001.pdf]

SUPPLEMENTARY MATERIALS

Title: A Multi-Modal MRI Analysis of Cortical Structure in Relation to Gender Dysphoria, Sexual Orientation, and Age in Adolescents.

Authors: Skorska, M. N., Chavez, S., Devenyi, G. A., Patel, R., Thurston, L., Lai, M.-C., Zucker, K. J., Chakravarty, M. M., Lobaugh, N. J., & VanderLaan, D. P.

EXAMINATION OF DEMOGRAPHIC VARIABLES AS POTENTIAL COVARIATES

Additional analyses were conducted to characterize the sample in terms of demographic variables not presented in the main text, and to examine whether these variables should be used as statistical controls in the main analyses.

Measures

For measures where a mean is calculated, the mean was computed for those participants who responded to at least 75% of the items.

Ethnicity

Participants self-reported their ethnicity, choosing from the following: "Aboriginal," "Asian," "European," "African," "East Indian," "Latin," and/or "other." Multiple ethnicities could be selected, and participants were asked to specify if they selected "other." Participants were dichotomized into those who indicated only "European" or only specified "White" or "Canadian" (n = 27) versus all other ethnicities, including multiple ethnicities. Those indicating "Canadian" are grouped with the "White" and "European" majority based on researcher observation. Other ethnicities included "Aboriginal" (n = 2), "Asian" (n = 4), "East Indian" (n = 1), "Middle Eastern" (n = 1), "Latin" (n = 2), and multiple ethnicities (n = 10).

Parents' Education

Participants self-reported the education of their mother and father separately by indicating the highest grade completed or the highest degree each parent received. These data were dichotomized into two categories: high school or less versus some post-secondary.

Parents' Marital Status

Participants self-reported their parents' marital status by selecting one of the following options: married to each other, cohabitating with each other, separated, divorced, single, or other (please specify). Participants also indicated the number of years and months that the father has not been living at home (if the father was not living at home). Responses were dichotomized into two categories: parents who were married/cohabitating and those who were not.

Wechsler Intelligence Subtests

Selected subtests from the Wechsler Intelligence Scale for Children (version V; [74]) were administered to participants 12 to 16 years old, and from the Wechsler Adult Intelligence Scale (version IV; [73]) to the 17-year-old participants. Four subtests covering verbal comprehension and visual spatial domains were completed and scaled scores were reported. For the verbal comprehension subtests, the vocabulary and either similarities or comprehension subtests were administered. For two participants, both similarities and comprehension were completed and the similarities scaled score was used. A verbal comprehension scaled score was computed by averaging the two verbal subtest scores (absolute range: 1-19; a score of 10 represents the 50th percentile). From the visual spatial domain, participants completed block design and either visual puzzles or object assembly (from the Wechsler Intelligence Scale for Children, version III; [72]). A visual spatial scaled score was computed by averaging the two visual spatial subtest scores (absolute range: 1–19).

Personal Development Scale and Menstrual Cycle

Pubertal stage in reference to sex assigned at birth was self-reported using the Personal Development Scale (PDS; [75]), which shows good reliability and validity. Participants reported the presence of five (cisgender boys) or four (cisgender girls and gender dysphoria assigned female at birth (GD AFAB)) secondary sex characteristics using a 4-point scale ranging from "no development" to "development has already completed." Participants reported on a growth spurt, skin changes (e.g., pimples), growing hair on their body in places other than their head (e.g., under arms, on/around genitals), growing hair on their face (boys), a change in their voice (boys), and breast growth (girls/GD AFAB). Cronbach's alpha was 0.95 for the boys and 0.70 for the girls/GD AFAB. A mean was calculated from these items where higher scores represented more advanced pubertal development.

Cisgender girls and GD AFAB were also asked whether they had started menstruating. All AFAB participants (n = 33) answered affirmatively. Participants were further asked about the regularity of their menstrual periods and only two (out of four possible) answers were chosen by participants: "My menstrual periods are not regular (some months they occur and some months they do not occur, the amount of and number of days of bleeding is very different from month to month)" and "Very regularly (every month with approximately 2-6 days of bleeding)." A dichotomous menstrual cycle regularity variable was created to represent participants with regular cycles versus participants with irregular cycles.

Medication Use

Participants completed a medication log of all medications they were currently taking, the dosage, and any side effects. Eight participants were taking one (n = 6) or two (n = 2) medications. Types of medication included SSRIs (Prozac, Zoloft), stimulants (Concerta, Adderall, Ritalin), antipsychotic medication (Risperidone), and birth control pills (Seasonale,

Micronor). A dichotomous variable was created representing those not taking any medication (including one participant who was taking Cold FX, a natural health product) versus those taking any medications.

Youth Self-Report

The Youth Self-Report (YSR) is a 118-item self-report questionnaire designed for youth between the ages of 11 and 18 years to assess general psychopathology [76-78]. Items were rated on a 3-point scale ranging from 0 to 2 for frequency of occurrence. Potential behavior problems were based on 102 of the items, which are used to index the degree of internalizing, externalizing, and total behavior problems, and the 16 other items reflect socially desirable behaviors ("filler" items that are not scored). Participants completed one of two versions of the YSR (1991 and 2001); the 1991 version contains two gender-specific items (Item 5: "I act like the opposite sex" and Item 110: "I wish I were of the opposite sex"), and the 2001 version contains one gender-specific item (Item 110). These items, along with any other responses indicating challenges related to gender questioning or dysphoria, were scored as 0s to avoid any artificial inflation of the general behavioral problem indices. *T* scores for the internalizing problems, externalizing problems, and total problems factors were used. Also, dichotomous variables for internalizing problems, externalizing problems, and total problems were calculated, representing whether the *T* scores were in the clinical range (>90th percentile; *T* score greater than 63) or not.

Statistical Analyses

Using SPSS version 27, group differences on continuous demographic variables were examined with one-way analyses of variance (ANOVA). In the presence of a significant omnibus effect, post hoc comparisons were conducted with least significant difference (LSD) tests. For

categorical variables, Fisher's exact tests were conducted with follow-up Bonferroni-corrected *z*-tests that compare column proportions in the presence of a significant omnibus effect. A two-tailed critical *p*-value of 0.05 was used.

Correlations between each variable and the 228 surface area, cortical thickness, and T1 ROIs were conducted. Bonferroni corrections for multiple tests were utilized for each demographic variable (adjusted *p*s = 0.05/228 = 0.000219).

Results: Group Differences

For all continuous variables, there were no extreme deviations from normality based on skewness and kurtosis values, which were less than |2|.

One-way ANOVAs for verbal comprehension index, visual spatial index, PDS score, and externalizing problems *T* score indicated no significant between-group differences (see Table S1). There was a significant difference for the internalizing problems *T* score such that the GD AFAB group scored significantly higher than both the cisgender girls (mean difference [MD] = 7.56, *SE* = 3.47, *p* = 0.035) and cisgender boys (MD = 16.05, *SE* = 3.64, *p* < 0.001), and the cisgender girls scored significantly higher than the cisgender boys (MD = 8.49, *SE* = 3.54, *p* = 0.021). There was also a significant difference for total problems *T* score such that the GD AFAB group (MD = 12.65, *SE* = 3.27, *p* < 0.001) and the cisgender girls (MD = 7.30, *SE* = 3.17, *p* = 0.026) scored significantly higher than the cisgender boys. The GD AFAB group did not score significantly higher than the cisgender girls (MD = 5. 35, *SE* = 3.12, *p* = 0.093).

Table S1. Descriptive statistics for additional demographic variables.

	Cisgender Boys	GD AFAB	Cisgender Girls	*F* (*df*) or Fisher's Exact Test	*p*
n[a]	14	16	17		
Ethnicity, *n* (%)					

"European"/"White"	6 (42.9)	10 (62.5)	11 (64.7)	1.73	0.468
Other	8 (57.1)	6 (37.5)	6 (35.3)		
Mother's education, *n* (%)					
High school or less	0	4 (25)	1 (5.9)	4.36	0.109
Any post-secondary	13 (92.9)	11 (68.8)	14 (82.4)		
n	13	15	15		
Father's education, *n* (%)					
High school or less	1 (7.1)	6 (37.5)	3 (17.6)	3.46	0.201
Any post-secondary	11 (78.6)	9 (56.3)	11 (64.7)		
n	12	15	14		
Parent's marital status, *n* (%)					
Married/living together	14 (100)	7 (43.8)	15 (88.2)	14.01	< 0.001
Other	0	9 (56.3)	2 (11.8)		
Verbal Comprehension					
M	13.25	11.53	11.91	1.95 (2, 43)	0.155
SD	2.21	2.18	2.94		
Range (1-19)[b]	9-17.50	6.5-14.5	5-17		
n	14	16	16		
Visual Spatial					
M	11.93	10.41	11.22	1.34 (2, 43)	0.272
SD	2.11	3.01	2.38		
Range (1-19)[b]	7.5-15.5	4-15	7.5-16.5		
n	14	16	16		
Personal Development Scale					
M	2.86	3.28	3.13	0.66 (2, 44)	0.206
SD	0.78	0.59	0.53		
Range (1-4)[b]	1.20-4.00	2.00-4.00	2.25-4.00		
Menstrual Cycle Regularity, *n* (%)					
Regular	-	14 (87.5)	10 (58.8)	-	0.118
Irregular	-	2 (12.5)	7 (41.2)		
Medication Use, *n* (%)					
None	14 (100)	8 (50)	17 (100)	16.05	< 0.001
1-2	0	8 (50)	0		
YSR: Internalizing Problems *T* Score					
M	49.21	65.27	57.71	9.72 (2, 43)	< 0.001
SD	10.74	10.36	8.38		
Range (27 or 30-100)[b,c]	35-66	49-84	45-80		
n	14	15	17		
YSR: Internalizing Problems Clinical Range, *n* (%)					
Non-clinical	13 (92.9)	6 (37.5)	13 (76.5)	9.62	0.008
Clinical	1 (7.1)	9 (56.3)	4 (23.5)		
n	14	15	17		
YSR: Externalizing Problems *T* Score					
M	51.79	58.60	54.47	1.84 (2, 43)	0.172

SD	9.60	9.84	9.59		
Range (29-100)[b,c]	30-63	38-78	34-71		
n	14	15	17		
YSR: Externalizing Problems Clinical Range, *n* (%)					
Non-clinical	14 (100)	11 (68.8)	15 (88.2)	4.17	0.115
Clinical	0	4 (25)	2 (11.8)		
n	14	15	17		
YSR: Total Problems *T* Score					
M	50.29	62.93	57.59	7.53 (2, 43)	0.002
SD	8.85	8.49	9.00		
Range (26-100)[b,c]	32-62	49-81	42-76		
n	14	15	17		
YSR: Total Problems Clinical Range, *n* (%)					
Non-clinical	14 (100)	6 (37.5)	13 (76.5)	13.27	0.001
Clinical	0	9 (56.3)	4 (23.5)		
n	14	15	17		

Note. YSR = Youth Self-Report.
[a]This is the full sample size for each group. If there were missing data, the sample size is indicated with the relevant variable.
[b]Indicates absolute range.
[c]A *T* score above 63 is considered to be in the clinical range. For Internalizing *T* scores, the lower bound *T* score is 27 for birth-assigned females and the lower bound *T* score is 30 for birth-assigned males.

Fisher's exact tests for ethnicity, education of mother, education of father, externalizing problems in the clinical range, and regularity of menstrual cycle indicated these variables were not related to group (see Table S1). There was a significant association between marital status and group. The GD AFAB group was significantly more likely to have parents who were separated/widowed/divorced than married/cohabitating, the cisgender boys were significantly more likely to have parents who were married/cohabitating than separated/widowed/divorced, and there was no significant difference between the two categories related to marital status within cisgender girls. Medication use was also related to group. The GD AFAB participants were significantly more likely to be taking medication than not taking medication, whereas the cisgender boys and girls were significantly more likely to be not taking medication than taking medication, which is not surprising given mental health diagnoses were an exclusion criterion for

cisgender participants. Variables representing whether internalizing problems and total problems were in the clinical range were both associated with group. For both, the GD AFAB group was significantly more likely to have a clinical flag than a non-clinical flag, the cisgender boys were significantly more likely to have a non-clinical flag than a clinical flag, and there was no significant difference between clinical or non-clinical flag within cisgender girls.

Results: Correlations with ROIs

The full correlation table can be found in the Excel file titled "SkorskaetalGDBrainPubCorrelationsDemoROIsN47.xlsx" [47]. Table S2 provides a summary of the number of significant correlations and the number of significant correlations after Bonferroni correction. Also, the largest correlation is provided, including the ROI for which this occurred. After correction for multiple comparisons, there were no significant correlations between any of the demographic variables and the ROI data, including demographic variables that showed significant group differences (i.e., internalizing problems *T* score and clinical flag, total problems *T* score and clinical flag, parent marital status, and medication use).

Table S2. Summary of correlation analysis between demographic variables and ROI data across participants.

Variable	Total Number of Significant Correlations, $p < 0.05$	Total Number of Significant Correlations after Bonferroni Correction, $p < 0.000219$	Largest Correlation			
			r	*p*	*n*	ROI
Age	24	0	–0.47	0.001	47	Right Heschl Gyrus T1
GIDYQ-AA	12	0	0.44	0.002	47	Left Cuneus Surface Area
Ethnicity	13	0	–0.43	0.003	47	Right Temporal Pole Superior Temporal Gyrus T1
Mother Education	27	0	0.44	0.003	43	Left Calcarine Fissure and Surrounding Cortex Surface Area
Father Education	28	0	0.52	0.000467	41	Right Supramarginal Gyrus Surface Area
Parent Marital Status	11	0	–0.46	0.001	47	Left Calcarine Fissure and Surrounding Cortex Surface Area
Verbal Comprehension Index	25	0	0.51	0.000331	46	Left Gyrus Rectus Cortical Thickness
Visual Spatial Index	26	0	0.51	0.000284	46	Left Gyrus Rectus Surface Area
PDS	3	0	–0.37	0.012	46	Left Inferior Frontal Gyrus Triangular Part Surface Area
Menstrual Cycle Regularity	6	0	0.48	0.005	33	Right Temporal Pole Superior Temporal Gyrus Surface Area
Medication Use	5	0	–0.37	0.010	47	Left Insula Cortical Thickness
YSR: Internalizing Problems *T* Score	9	0	–0.46	0.001	46	Left Superior Frontal Gyrus Medial Orbital Cortical Thickness
YSR: Internalizing Problems Clinical Range	4	0	–0.36	0.014	46	Right Inferior Frontal Gyrus Orbital Part Cortical Thickness
YSR: Externalizing Problems *T* Score	12	0	–0.51	0.000305	46	Left Middle Occipital Gyrus Cortical Thickness
YSR: Externalizing	12	0	–0.37	0.011	46	Left Middle Frontal Gyrus Orbital Part

Problems Clinical Range						Cortical Thickness
YSR: Total Problems *T* Score	15	0	–0.47	0.001	46	Left Superior Frontal Gyrus Medial Orbital Cortical Thickness
YSR: Total Problems Clinical Range	19	0	–0.43	0.003	46	Left Superior Frontal Gyrus Orbital Part Cortical Thickness

Note. ROI = region of interest, GIDYQ-AA = Gender Identity/Gender Dysphoria Questionnaire for Adolescents and Adults, PDS = personal development scale, YSR = Youth Self-Report.

SUPPLEMENTARY METHODS FIGURES

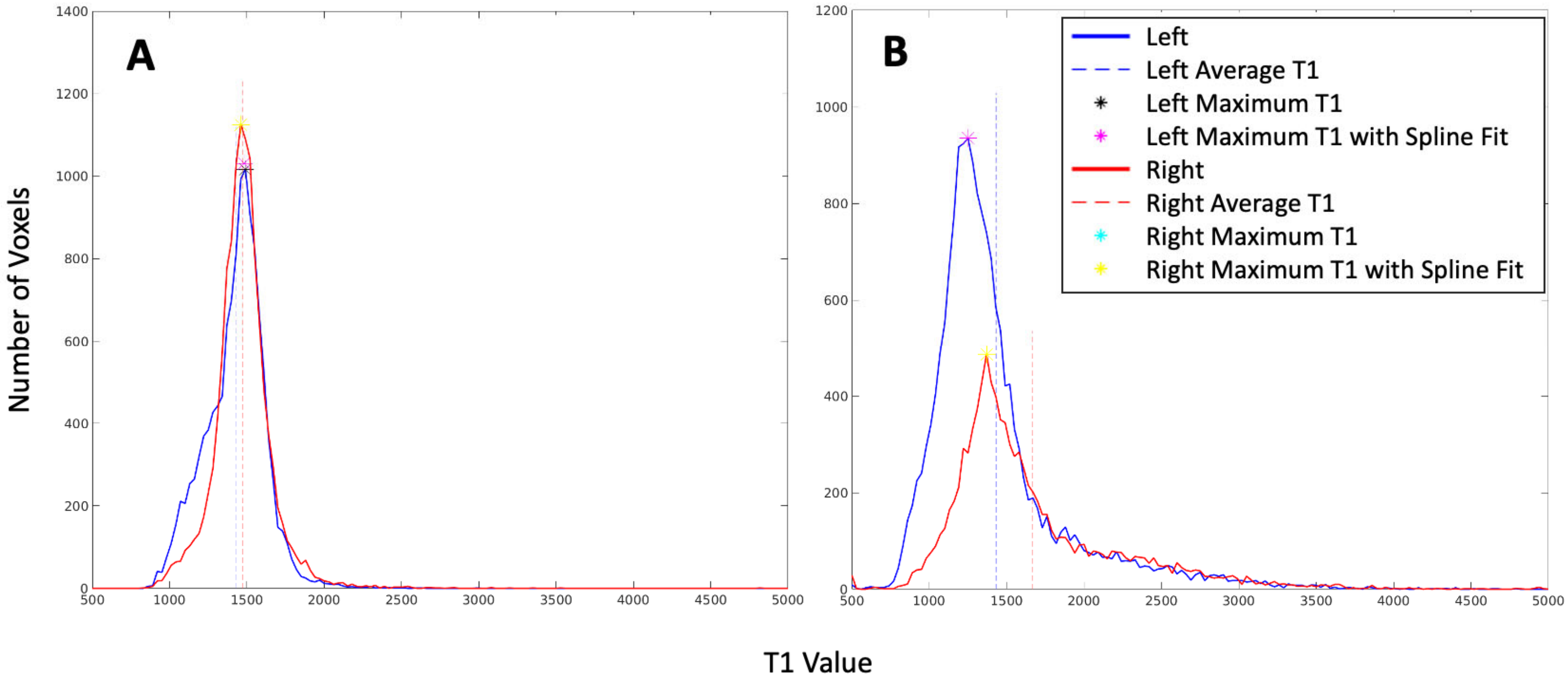

Supplemental Figure S1. Examples of T1 Histograms. Panel A. A T1 histogram from the left and right hemispheres of the middle occipital gyrus is shown. In this case, the distribution of T1 values was positively skewed. A spline function was fit to the histogram to determine the maximum T1 value (mode). **Panel B.** A T1 histogram from the left and right hemispheres of the insula is shown. Here, the distribution of T1 values was more symmetrical around the mean.

SUPPLEMENTARY PARTIAL LEAST SQUARES FIGURES

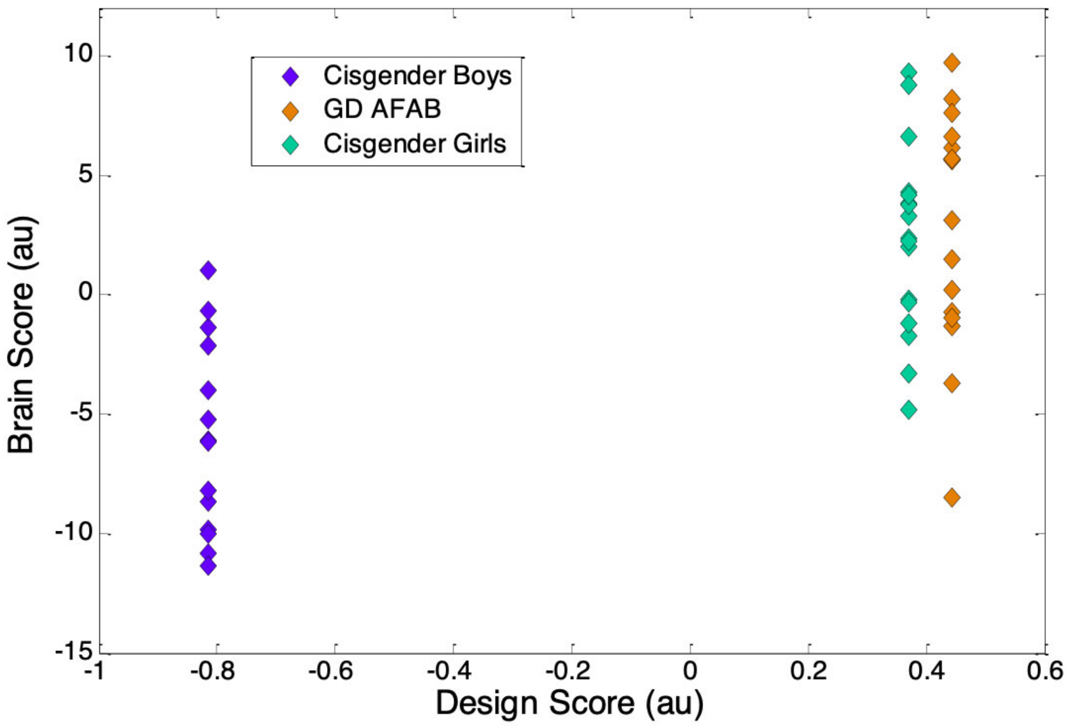

Supplemental Figure S2. Task PLS results (brain scores by design scores). Brain scores plotted as a function of design scores for cisgender boys (purple), GD AFAB (dark orange), and cisgender girls (green). These scores are also shown in Fig. 2, Panel A as bar plots with 95% CIs.

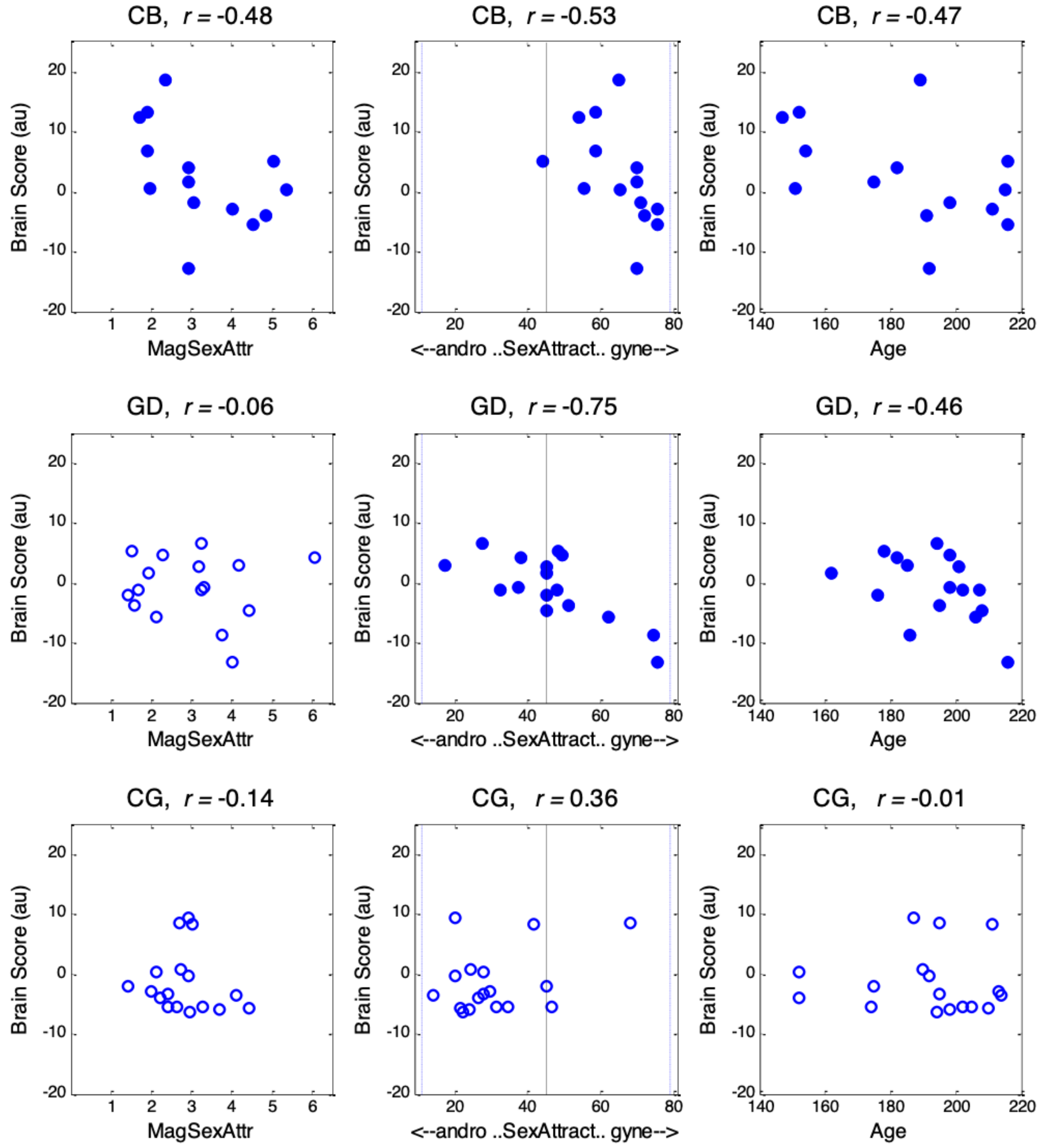

Supplemental Figure S3. Scatterplots showing the brain score-behavior correlations for all groups and behavioral measures. These correlations are also shown in Fig. 3, Panel A as bar plots with 95% CIs. Left column is the strength of sexual attraction (MagSexAttr). Center column is the direction of sexual attraction (SexAttr) with a range from exclusively androphilic (11°) to exclusively gynephilic (79°). The dotted line at 45°indicates ambiphilic or asexual and the range is indicated by the dot-dashed line. Right column is age (months). The strength of the brain–behavior correlation is indicated at the top of each plot, and stable correlations are indicated by filled markers. CB = cisgender boys, GD = GD AFAB, CG = cisgender girls.

REFERENCES

47. Skorska, M. N.; Chavez, S.; Devenyi, G. A.; Patel, R.; Thurston, L.T.; Lai, M.-C.; Zucker, K. J.; Chakravarty, M. M.; Lobaugh, N. J.; VanderLaan, D. P. Data from: A multi-modal MRI analysis of cortical structure in relation to gender dysphoria, sexual orientation, and age in adolescents. *Scholar's Portal Dataverse* **2020**. doi:10.5683/SP2/B3HMPQ
72. Wechsler, D. *Wechsler intelligence scale for children (Third ed.).* San Antonio, TX: The Psychological Corporation **1991**.
73. Wechsler, D. *Wechsler adult intelligence scale (Fourth ed.).* San Antonio, TX: Pearson **2008**.
74. Wechsler, D. *Wechsler intelligence scale for children (Fifth ed.).* San Antonio, TX: The Psychological Corporation **2014**.
75. Petersen, A. C.; Crockett, L.; Richards, M.; Boxer, A. A self-report measure of pubertal status: Reliability, validity, and initial norms. *J. Youth Adolesc.* **1988**, *17*, 117–133. doi:10.1007/BF01537962
76. Achenbach, T. M. *Manual for the Youth Self-Report and 1991 Profile.* Burlington, VT: University of Vermont Department of Psychiatry **1991.**
77. Achenbach, T. M.; Edelbrock, C. *Manual for the Youth Self-Report and Profile.* Burlington, VT: University of Vermont Department of Psychiatry **1986.**
78. Achenbach, T. M.; Rescorla, L. A. *Manual for the ASEBA School-Age Forms & Profiles.* Burlington, VT: University of Vermont, Research Center for Children, Youth, & Families **2001.**
